# The Glycosylated Rv1860 Protein of *Mycobacterium tuberculosis* Inhibits Dendritic Cell Mediated TH1 and TH17 Polarization of T Cells and Abrogates Protective Immunity Conferred by BCG

**DOI:** 10.1371/journal.ppat.1004176

**Published:** 2014-06-12

**Authors:** Vijaya Satchidanandam, Naveen Kumar, Rajiv S. Jumani, Vijay Challu, Shobha Elangovan, Naseem A. Khan

**Affiliations:** 1 Department of Microbiology and Cell Biology, Indian Institute of Science, Bangalore, Karnataka, India; 2 National Tuberculosis Institute, Bangalore, Karnataka, India; Portland VA Medical Center, Oregon Health and Science University, United States of America

## Abstract

We previously reported interferon gamma secretion by human CD4^+^ and CD8^+^ T cells in response to recombinant *E. coli*-expressed Rv1860 protein of *Mycobacterium tuberculosis* (MTB) as well as protection of guinea pigs against a challenge with virulent MTB following prime-boost immunization with DNA vaccine and poxvirus expressing Rv1860. In contrast, a Statens Serum Institute *Mycobacterium bovis* BCG (BCG-SSI) recombinant expressing MTB Rv1860 (BCG-TB1860) showed loss of protective ability compared to the parent BCG strain expressing the control GFP protein (BCG-GFP). Since Rv1860 is a secreted mannosylated protein of MTB and BCG, we investigated the effect of BCG-TB1860 on innate immunity. Relative to BCG-GFP, BCG-TB1860 effected a significant near total reduction both in secretion of cytokines IL-2, IL-12p40, IL-12p70, TNF-α, IL-6 and IL-10, and up regulation of co-stimulatory molecules MHC-II, CD40, CD54, CD80 and CD86 by infected bone marrow derived dendritic cells (BMDC), while leaving secreted levels of TGF-β unchanged. These effects were mimicked by BCG-TB1860His which carried a 6-Histidine tag at the C-terminus of Rv1860, killed sonicated preparations of BCG-TB1860 and purified H37Rv-derived Rv1860 glycoprotein added to BCG-GFP, but not by *E. coli*-expressed recombinant Rv1860. Most importantly, BMDC exposed to BCG-TB1860 failed to polarize allogeneic as well as syngeneic T cells to secrete IFN-γ and IL-17 relative to BCG-GFP. Splenocytes from mice infected with BCG-SSI showed significantly less proliferation and secretion of IL-2, IFN-γ and IL-17, but secreted higher levels of IL-10 in response to *in vitro* restimulation with BCG-TB1860 compared to BCG-GFP. Spleens from mice infected with BCG-TB1860 also harboured significantly fewer DC expressing MHC-II, IL-12, IL-2 and TNF-α compared to mice infected with BCG-GFP. Glycoproteins of MTB, through their deleterious effects on DC may thus contribute to suppress the generation of a TH1- and TH17-dominated adaptive immune response that is vital for protection against tuberculosis.

## Author Summary

Tuberculosis (TB), although recognized as an infectious disease for centuries, is still the leading cause of human deaths, claiming a million lives annually. Successful control of TB, either through drugs or effective preventive vaccines has not been achieved despite decades of research. We have studied the role for mannosylated protein Rv1860 of MTB in interfering with the early response of dendritic cells, which belong to the host's innate immune arsenal, to this mycobacterium. We were able to show that incorporating the gene coding for Rv1860 of MTB into the safe vaccine strain BCG resulted in loss of BCG's protective ability in the guinea pig animal model. Using primary mouse bone marrow derived dendritic cells *in vitro* as well as spleen dendritic cells from infected mice, we show in this study that exposure to mannosylated Rv1860 leads to loss of dendritic cell functions such as cytokine secretion and T cell activation. This leads to defective downstream T cell responses to the mycobacteria. We suggest that altering or extinguishing the expression of such glycoproteins by mycobacteria may be a strategy for developing better vaccines against TB.

## Introduction

The scourge of tuberculosis which claimed close to a million non-HIV infected victims in 2011 worldwide [1] aided by multiple (MDR) and extremely drug resistant (XDR) strains [2] of the causative organism *Mycobacterium tuberculosis* (MTB), has entrenched itself in the human population in its latent form and is undisputedly one of the most dreaded human bacterial diseases. MTB employs multiple mechanisms to interfere with both the innate and adaptive arms of the vertebrate immune system. These include inhibition of (i) phagolysozome fusion within antigen presenting cells [3], (ii) maturation of human monocytes into DC [4], (iii) dendritic cell migration to secondary lymphoid organs [5] as well as antigen processing and presentation to T cells [6], [7]. In addition, MTB-infected macrophages, but not DC, prevented the development of a TH1-polarized T cell response [8].

The ability of the infected host to control infection by MTB depends on the capacity of the innate immune cells, primarily professional antigen-presenting cells such as DC and macrophages to prime an early and effective adaptive T cell response [9], [10]. The presence of numerous pattern recognition receptors (PRR) on DC that are linked to intracellular signaling pathways allows these specialized cells to readily perceive invading pathogens and upregulate surface co-stimulatory molecules as well as secrete inflammatory and regulatory cytokines [11], both of which have a crucial bearing on the subsequent development of T cell responses. It is therefore to be expected that a successful pathogen such as MTB would target this subset of cells to subvert the generation of effective host-protective immune responses.

While the presence of complex lipid and carbohydrate moieties such as lipoarabinomannan, mycolic acids, phenolic glycolipids, peptidoglycan, phosphatidyl inositol mannosides etc. on the mycobacterial cell surface has been recognized for a very long time, awareness of the existence of glycosylated proteins in prokaryotic organisms has only come about over the last couple of decades. The pathogenic nature of several bacteria that possess glycosylated proteins, such as *Mycobacterium* and *Clostridium* species, *Campylobacter jejuni*, *Treponema pallidum, Pseudomonas aeruginosa, Helicobacter pylori, Neisseria meningitis, N.gonorrhoea, and Streptococcus parasanguis* (reviewed in [12]) suggests a role for these glycoproteins in mediating virulence and/or pathogenicity of these organisms. *M. tuberculosis* codes for at least forty one glycoproteins based on mass spectrometric characterization of concanavalin-A (Con-A) binding proteins [13], [14]. The two secreted glycoproteins that have been well characterized, namely Rv1860 of MTB [15], *M. bovis* and BCG [16] and MPB83 of *M. bovis* [17] carry one to three mannose residues linked to each other by α1→2 and 1→3 glycosidic bonds on threonine residues, respectively.

The varied range of carbohydrate structures present in the MTB cell is dominated by the hexose mannose. Lipoarabinomannan (LAM) from MTB was originally credited with much of the subversion of host immunity effected by this pathogen, by binding to the carbohydrate receptors mannose receptor (MR) on human macrophages [18] and dendritic cell-specific intercellular adhesion molecule-3 grabbing non-integrin (DC-SIGN) on human dendritic cells (DC) [19]. Subsequent investigations pointed to significant contribution from other ligands carrying glycosylated structures from MTB [20], [21] such as glycoproteins. Early reports relied primarily on Con-A-binding as proof of protein glycosylation [22], [23] in mycobacteria. One of these glycoproteins, Rv1860, also referred to as 45/47 kDa culture filtrate protein or APA owing to the presence of repeating units of alanine-proline-alanine motifs, was first identified as a proline-rich culture filtrate protein capable of eliciting both a delayed type hypersensitive (DTH) [24] and an antibody response [25] in guinea pigs immunized with live, but not killed *Mycobacterium bovis* BCG. The MTB homolog coding for a 50–55 kDa, 325 amino acid long Rv1860 protein [26], was subsequently cloned and expressed both in *M. smegmatis* and *E. coli* [27]. Elegant analysis of the glycosylation moieties by proteolytic digestion of the purified 45 kDa culture filtrate-derived Rv1860 protein of MTB followed by mass spectrometry revealed the amino acid residues glycosylated to be threonines 10, 18, 27 and 277 and the attached carbohydrate to be single mannose, mannobiose, or mannotriose units strung together by α1→2 linkages [15]. Alteration of glycosylation of Rv1860 by expression in *M. smegmatis* as well as loss of glycosylation by enzymatic digestion or expression in *E. coli* resulted in reduced ability to elicit a DTH reaction in guinea pigs [16], [28]. Both 45 and 47 kDa species had lost their 39 amino acid long N-terminal signal sequence; while the 45 kDa species carried predominantly a single mannose per molecule, the 47 kDa protein was dominated by 6 to 9 mannose residues per molecule [16].

We had initially identified Rv1860 as a target of antibody responses in sputum positive pulmonary TB patients [29] and demonstrated the ability of live, but not killed bacilli to elicit an antibody response in guinea pigs against several MTB proteins including Rv1860 [30]. We subsequently reported that recombinant *E.coli*-expressed Rv1860 was a robust *in vitro* stimulator of both CD4^+^ and CD8^+^ T cells from healthy PPD-positive volunteers [31] and that Rv1860 expressed in eukaryotic systems including a DNA vaccine vector and poxvirus protected guinea pigs against a challenge dose of virulent MTB. The contrasting loss of BCG's protective efficacy upon expression of Rv1860 from a genomically integrated copy of the MTB-derived gene in BCG-TB1860 suggested subversion of innate immune cells by native glycosylated Rv1860. Since DCs possess multiple receptors to sense glycosyl moieties unique to pathogens, including mannose receptor, dectins and several C-type lectins including DC-SIGN, SIGNR1 to R4 and pulmonary surfactant proteins [32]–[35], we investigated the effect of MTB Rv1860 on functions of primary murine bone marrow-derived dendritic cells (BMDC).

## Materials and Methods

### Ethics statement

All experiments with animals were carried out in strict accordance with the Institutional Animal Ethics Committee-approved protocols of the Indian Institute of Science for mouse experiments (CAF/Ethics/220-2011 dated February 10, 2011) and National Tuberculosis Institute for guinea pig experiments (NTI-IAEC-2608-2610 dated 25 January, 2005). All protocols adhered to the guidelines provided by the “Committee for the Purpose of Control and Supervision of Experiments on Animals” (CPCSEA), a statutory body of the Government of India, Ministry of Social Justice and Empowerment.

### Plasmid construction

We constructed the plasmids pDK-Hyg-Rv1860, pDK-Hyg-GFP and pDK-Hyg-Rv1860-6His (Figure S1 in Text S1) expressing the Rv1860 gene of H37Rv, the control gene GFP and Rv1860 with a C-terminal 6× Histidine tag, respectively as described in Supplementary Methods in Text S1.

### Mycobacterial strains and culture

BCG-SSI-1331 from Statens Serum Institute was grown in Middlebrook 7H9 liquid medium (Difco, Detroit, USA) supplemented with 10% albumin/dextrose/catalase (Difco), 0.2% glycerol, and 0.05% Tween 80. Aliquots of mid log phase cultures were frozen at −85°C and viable bacteria were enumerated by plating serial dilutions on 7H10 agar plates supplemented with 10% oleic acid albumin dextrose catalase (Difco) and 0.2% glycerol (1 A_600 nm_ = 10^9^ colony forming units [cfu]/ml). To construct BCG strains expressing GFP (BCG-GFP), and *M. tuberculosis* Rv1860 without and with the C-terminal 6× Histidine tag, (BCG-TB1860 and BCG-TB1860His; Table 1), *M. bovis* BCG-SSI was transformed with pDK-Hyg-GFP, pDK-Hyg-Rv1860 and pDK-Hyg-Rv1860-6His, respectively by electroporation at 2.5 kV, 200 µF and 1000 Ohms in a BIORAD Gene Pulser Xcell electroporator and selected on 7H10 plates supplemented with 75 µg/ml hygromycin. Single colonies were grown in 7H9 broth supplemented with 75 µg/ml hygromycin and the presence of the endogenous Rv1860 homolog as well as the integrated Rv1860 and GFP were verified by PCR amplification from genomic DNA as described in legend to Figure S2 in Text S1. Expression from endogenous and inserted copies of Rv1860 protein in the BCG strains was quantitated by Western blotting lysates of the BCG strains with mouse anti-Rv1860 serum (Supplementary Methods in Text S1) or mouse anti-6His antibody (BD Biosciences), followed by chemiluminescence detection, with ribosome release factor serving as loading control.

**Table 1:** Description of strains and reagents used in the study.

Name	Description
BCG-GFP	Copenhagen strain obtained from Statens Serum Institute (BCG-SSI) expressing GFP from the gene driven by the mycobacterial *hsp* promoter, integrated at the genomic phage L5 integration site.
BCG-TB1860	BCG-SSI expressing the MTB Rv1860 gene driven by the *hsp* promoter, integrated at the genomic phage L5 integration site.
BCG-TB1860His	BCG-SSI expressing the MTB Rv1860 gene with a C-terminal 6× Histidine tag, driven by the *hsp* promoter, integrated at the genomic phage L5 integration site.
g1860	Purified glycosylated native Rv1860 protein from *M. tuberculosis* (NR-14862) obtained from Biodefense and Emerging Infections Resources, NIAID, NIH, USA.

### Immunoprecipitation and western blotting

Western blotting of bacterial lysates obtained by sonication of cell pellets as described below, utilized 50 µg protein per lane of the polyacrylamide gel. For immunoprecipitation, lysates of BCG strains (400 µg protein) and culture filtrates (10 ml from cultures with A_600 nm_ = 0.7) were incubated with 10 µl of rabbit serum raised to recombinant Rv1860 protein (described in Supplementary methods in Text S1) or 2.5 µg mouse monoclonal antibody specific to 6×Histidine (Cell Biolabs) coated on Protein A-Sepharose beads in binding buffer (50 mM Tris, pH 8.0, 0.5 M NaCl, 0.1% deoxycholate, 0.1% sodium lauryl sulphate, 0.2% Nonidet P40, and 0.05% Tween-20) overnight at 4°C. Beads were washed thrice with binding buffer and eluted by boiling with Laemmli sample buffer for 3 min. Binding and washing of BCG lysates and culture filtrates to Con-A-sepharose beads (GE Healthcare) was carried out in 20 mM Tris-HCl pH 7.4, 0.5 M NaCl, 1 mM MnCl_2_, 1 mM CaCl_2_. Beads were eluted with 0.1 M borate buffer, pH 6.5. Samples were electrophoresed on SDS-10% PAGE, transferred to nitrocellulose membranes and probed with appropriate mouse anti-Rv1860 serum, mouse monoclonal antibody specific to 6× Histidine (BD Pharmingen clone F24-796) or Con-A-HRP as described in Figure legends followed by chemiluminescence detection.

### BMDC and peritoneal macrophage culture

8 to 10 week old female BALB/c mice were euthanized and femurs and tibia recovered by dissection. Following removal of muscle and connective tissues, bones were carefully cut at the ends to expose the marrow which was flushed out using plain Iscove's Modification of Dulbecco's Minimum Essential Medium (IMDM). Bone marrow cells were cultured in IMDM-10% FBS containing 2 mM β-mercapto-ethanol essentially according to standard published protocols [8] with 50 U/ml murine rGM-CSF (R&D Systems, Minneapolis, MN) in the absence of antibiotics. 50% of the culture medium was removed and replenished every two days. Non-adherent cells were harvested on day 7 and analysed by flow cytomtery using antibodies against MHC-II, CD80, CD86, CD54 and CD40 to confirm that >90% of the cells represented immature dendritic cells which were then used for all experiments.

Peritoneal macrophages were obtained from female BALB/c mice by washing the peritoneal cavity with IMDM. 2×10^5^ cells were seeded per well of a 96 well plate. Adherent cells obtained following washing to remove the non-adherent population were infected at 1 multiplicity of infection (MOI).

### Infection of BMDCs with *M. bovis* BCG strains

DCs were infected with BCG strains mentioned above at an MOI of 5 for all experiments unless otherwise mentioned. MOI of one was used to assess survival of BCG strains within DC by colony enumeration of infected DC lysates. Mycobacterial cultures were concentrated to give 100 A_600 nm_ units (10^11^ cfu/ml) and sonicated for 10 min. using 30 sec pulses in a Branson Model 450 cup horn sonifier. Sonicates were plated on 7H10 agar plates to confirm absence of live cells and sonicate comparable to 5 MOI was added to BMDC cultures. Purified glycosylated native Rv1860 protein (g1860) from *M. tuberculosis* (NR-14862) obtained from Biodefense and Emerging Infections Resources, established by the National Institute of Allergy and Infectious Diseases (NIAID) and purified recombinant *E. coli*-expressed MTB Rv1860 (Table 1) were each used at 10 ng per well of a 96 well plate, a concentration that was determined to be comparable to that delivered by infection at 5 MOI based on Western blotting serial dilutions of the purified proteins and BCG-TB1860 lysates with mouse anti-Rv1860 followed by quantitation of chemiluminescence signals. LPS from *E. coli* (Sigma) was used at 0.1 µg/ml.

### Infection of mice with BCG strains

For *in vitro* recall responses of splenocytes, female BALB/c mice were infected intraperitoneally with 5×10^8^ cfu of BCG-SSI. Three weeks later, spleens were harvested and used for *in vitro* stimulation assays described below. To study the effect of MTB-Rv1860 on spleen DC *in vivo*, we infected mice with 10^7^ cfu of the two BCG strains BCG-GFP or BCG-TB1860. Production of cytokines 1L12p40, IL-2 and TNF-α as well as up regulation of MHC class II on spleen DC *ex vivo* was measured 12 hrs later by flow cytometry using the gating strategy shown in Figure S4.

### T cell polarization and recall responses

For MLR studies, BMDC from eight weeks old female BALB/c mice were infected at an MOI of 5 with BCG strains for 24 hours, washed, gamma irradiated (15 Gray) and cultured in triplicate wells with non-adherent splenocytes from female C57/Black6 mice for 72 hours at DC: splenocyte ratios of 1∶10, 1∶20 and 1∶100 in 96 well culture dishes. For syngeneic polarization studies, the above mentioned infected BMDC were co-cultured with 5×10^5^ splenocytes per well from BALB/c mice infected 3 weeks previously with 5×10^8^ BCG-GFP or BCG-TB1860, at DC: splenocyte ratio of 1∶20 for 72 hrs. Supernatants were collected for cytokine measurements and cells were pulsed with 0.5 µCi ^3^H-thymidine for 16 hrs. Cells were harvested in a Perkin Elmer Filtermate harvester and counted in a LKB Rack Beta liquid scintillation counter (Model 1209, LKB-Wallac, Turku, Finland). Con-A at 5 µg/ml was included as positive control. Irradiated DC alone incorporated between 40 and 90 cpm while T cells alone incorporated between 280 to 970 cpm. Con-A stimulation resulted in incorporation of 24,000 to 43,000 cpm in multiple experiments.

To assess the effect of BCG strains on *in vitro* recall responses of immunized mice, splenocytes from mice immunized with 5×10^8^ cfu of BCG-SSI three weeks before, were cultured at 5×10^5^ cells per well in triplicate wells of a 96 well plate for 72 hrs in the presence of 20 MOI BCG-GFP or BCG-TB1860. Supernatants were collected for cytokine measurements. Con-A at 5 µg/ml was included as positive control.

### Antibodies, FACS staining, and acquisition

Staining for surface markers on BMDC was done by resuspending up to 1×10^6^ cells in 100 µl FACS buffer (PBS supplemented with 1% heat-inactivated FBS, 0.1% NaN3, and 1 mM EDTA) containing combination of anti-CD11c FITC (cloneN418) with one of the following antibodies, all from eBioscience: anti-CD40 PE (1C10), anti-CD86 PE (PO3.1) anti-CD80 PE (16-10A1), anti-MHC class II PE (M5/114.15.2), anti-CCR7 PE (4B12) for 30 min at 4°C (or at 37°C for CCR7). Splenocytes from mice immunized twice 3 weeks apart with 5×10^8^ BCG-SSI were obtained one week after the second immunization. 6×10^6^ million splenocytes seeded in 3 ml per 35 mm dish were infected at an MOI of 20 for 36 hrs with BCG-GFP or BCG-TB1860. 10 µg/ml brefeldin and 0.75 µM monensin were added for the final 26 hrs of culture. Harvested splenocytes were washed once with PBS-0.05% azide, permeabilized in 500 µl of PBS-0.05% N_3_-1% BSA with 0.1% saponin for 30 min. and intracellular IL-17 was detected using an antibody cocktail made up of CD3-FITC (clone 145-2C-11), CD4-PE (clone GK1.5), CD25-PE-Cy7 (clone PC 61.5) from BD Biosciences and IL-17-APC (clone eBio17B7) for 30 min on ice. IL-17 producing cells within the CD3-high, CD4-high and CD25-low population were enumerated. Detection of cytokines produced by mouse spleen DC *ex vivo* following 12 hr infection with 10^7^ cfu of the BCG strains was carried out using 2×10^6^ fresh splenocytes in the absence of *in vitro* re-stimulation as above using an antibody cocktail consisting of CD11c-fluorescein isothiocyanate (FITC), MHC class II-PE/IL-12-phycoerythrin (PE) (clone C17.8), IL-2-PECy7 (clone JES6-5H4), anti-mouse TNF-α-phycoerythrin-cyanine-7 (PECy7) (clone MP6-XT22) F4/80-Alexa 647 (clone BM8), anti-mouse CD19-Alexa647 (clone 1D3), anti-mouse CD3-allophycocyanain (APC) (clone 145-2C11), anti-mouse Gr-1-APC (clone RB6-8C5) obtained from BD Biosciences or eBiosciences. Cells were washed twice and fixed in 2% paraformaldehyde for 15 min. at 4°C. Data were acquired using a BD FACS Canto flow cytometer. The APC/Alexa647-high T and B cells, macrophages and neutrophils were gated out and cytokine production by CD11c-high DC within the APC-low population was enumerated. Data were analysed using FlowJo software (Treestar).

### Immunization and challenge of guinea pigs

Outbred albino guinea pigs weighing 300 to 350 g bred at the National Tuberculosis Institute since 1979 and referred to as NTI-bred strain were used. Groups of eight animals were immunized intradermally with one of the following: (*i*) BCG, 10^6^ CFU one time only (*ii*) BCG-Rv1860, 10^6^ CFU one time only. Animals were challenged via the intramuscular route in the thigh muscle 4 weeks after immunization with 2×10^5^ viable MTB strain NTI64719 [29]. This challenge dose was sufficient to cause weight loss followed by death during weeks 7 to 8 post challenge in all control animals [36]. Animals were allowed free access to standard laboratory food and water for 6 weeks after which spleens were removed aseptically, homogenized and cultured for CFU of MTB as described [37], [38]. Humane endpoints for euthanasia established by the institutional animal ethics committee (NTI), which included being moribund or exceeding acceptable weight loss and/or being affected in their respiratory rate, were strictly followed.

### Intracellular survival of BCG strains within DC and peritoneal macrophages

7-day GM-CSF-differentiated DCs or mouse peritoneal macrophages were infected with BCG-GFP and BCG-TB1860 at an MOI of 1 for four hours. Following 3 washes to remove excess bacteria, remaining extracellular bacteria were killed by treatment with 50 µg/ml gentamicin for 1 hr. Infected DCs were lysed immediately or 72 h later, serially diluted, and colonies appearing on 7H10 agar plates were counted after 20 days.

### Quantitation of cytokines by ELISA

IFN-γ, IL-2, IL-4, IL-10, IL-12p40 and p70, TNF-α and TGF-β were quantitated by ELISA using commercially available antibody pairs (Duo set, R&D Systems) according to manufacturer's instructions. The lower limit of detection for all cytokines was 15 pg/ml.

### Statistical analysis

Comparisons between groups of guinea pigs were performed using *t* tests on log-transformed data. Statistical comparisons of cytokines, stimulation indices, surface marker expression on BMDC and cytokine-producing T cells were carried out using non-parametric Student's t test in GraphPad Prism for Windows (GraphPad Software, San Diego California USA). *P* values less than 0.05 were considered statistically significant.

## Results

### Construction and characterization of recombinant BCG strains

**Figure 1:**
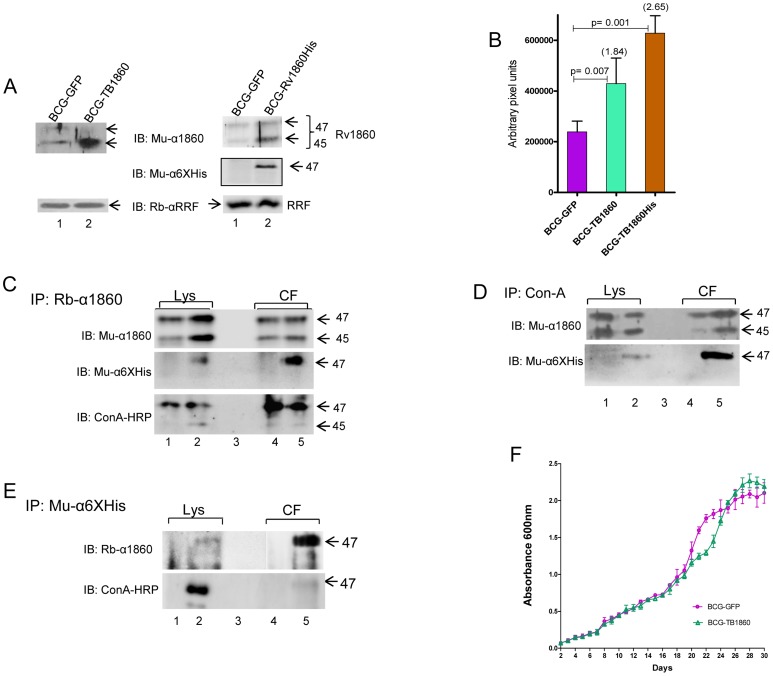
Characterization of recombinant BCG strains expressing MTB Rv1860. (A) Quantitation of Rv1860 expression. 4 µg protein from each of the strains BCG-GFP, BCG-TB1860 and BCG-TB1860His was electrophoresed in a SDS-10% PAGE, transferred to nitrocellulose membrane and probed using a mouse antiserum specific to Rv1860 (top panels), or rabbit antiserum to MTB ribosome release factor (RRF; bottom panels); 50 µg lysates were probed with mouse anti-6×Histidine antibody (middle panel on the right). Bands were detected by chemiluminescence. The 47 and 45 kDa forms of Rv1860 are shown by arrows as also the RRF. (B) Quantitation of expression of Rv1860 in the 2 strains shown in (A). Mean of 3 replicate experiments was used for quantitation of pixels obtained from chemiluminescence detection (C, D and E) Immunoprecipitation of 400 µg lysates (Lys; lanes 1 and 2) and 20 ml culture filtrates (CF; lanes 4 and 5) of BCG-GFP (lanes 1 and 4) and BCG-TB1860His (lanes 2 and 5) using rabbit anti-Rv1860 serum (C), Con-A-sepharose beads (D) and mouse anti-6×Histidine (E). Samples electrophoresed on a SDS-10% PAGE were transferred to nitrocellulose membrane and western blotted with mouse anti-Rv1860 (top panels, 10% in C and D and 50% in E), mouse anti-6×His antibody (45%, middle panel in C and lower panel in D) or Con-A-HRP (lower panel, 45% in C and 50% in E). The lysate samples in E required much longer exposure time than the CF and were hence developed separately (F) Growth curves for BCG-GFP (magenta, circles) and BCG-TB1860His (green, triangles) grown in 7H9 broth cultures.

BCG-SSI (1331) obtained from the Statens Serum Institute, Copenhagen, Denmark was electroporated with plasmids pDK-Hyg-TB1860, pDK-Hyg-Rv1860-6His and pDKHyg-GFP (Figure S1 in Text S1) and selected on hygromycin agar plates to obtain BCG-TB1860, BCG-TB1860His and BCG-GFP, respectively (Table 1). Genomic DNA from the parent and recombinant strains were analyzed by polymerase chain reaction using primers shown in Table 2 to amplify the endogenous Rv1860 gene as well as the integrated exogenous copies of Rv1860 and GFP genes as described in the Methods section. As expected, the parent BCG-SSI strain carried only the endogenous Rv1860 gene while BCG-TB1860, BCG-TB1860His and BCG-GFP carried in addition, the exogenous Rv1860, Rv1860-6×His and GFP genes, respectively (Figure S2 in Text S1). Western blotting with mouse serum specific to Rv1860 revealed a 1.84 fold and 2.65 fold increase in expression of Rv1860 in the BCG-TB1860 and BCG-TB1860His strains, respectively (Figure 1, A and B).

**Table 2:** Sequences of oligonucleotide primers used in the study.

Sl No	Primer name	Sequence
1.	FP1	5′GTGACCATGGCTAGCCATCAGGTGGACCCCAACTTGACACG 3
2.	RP1	GTGAGGATCCTCAGGCCGGTAAGGTCCGCTGCGGTGT
3.	FP2	5′ TTTTTTTTGGATCCATGCATCAGGTGGACCCCAACTTG 3′
4.	RP2	5′TTTTTTTTGTTAACTCAGTGGTGGTGGTGGTGGTGGGCCGGTAAGGTCCGCTGC 3′
5.	FP3	5′ TAACACGGTAGGTTCTTCGCC 3′
6.	RP3	5′GCTCCCATGGTCAGGCCGGTAAGGTCCGCTGC 3′
7.	FP4	5′TTTTTTTTGGATCCATGCATCAGGTGGACCCCAACTTG 3′
8.	RP4	5′ GGG GGT TTCTTGTCAGTACGC 3′
9.	FP5	5′ TTTTTTTTGGAGAAGAACTTTTCACTGGAGT 3′
10.	FP6	5′GCTCCCATGGTCAGTGGTGGTGGTGGTGGTGGGCCGGTAAGGTCCGCTGC 3′

To determine whether the recombinant Rv1860 protein expressed by BCG-TB1860 was mannosylated and secreted similar to the endogenous BCG homologue, we utilized BCG-TB1860His, carrying the C-terminally 6× Histidine-tagged Rv1860 gene. Western blotting with a mouse anti-6×Histidine antibody detected the 47 kDa 6×Histidine tagged protein in lysates from BCG-TB1860His but not in lysates from BCG-GFP (Figure 1A). Immunoprecipitates of cell lysates and culture filtrate proteins from the BCG-GFP and BCG- TB1860His strains with rabbit antiserum to Rv1860 protein or with Con-A-sepharose, when analyzed by Western blotting, contained the 47 kDa protein detected by anti-6×Histidine antibody only in BCG-TB1860His (Figure 1, C and D, respectively). The 45 kDa form of Rv1860, previously demonstrated to represent the mono-mannosylated species [16], was consistently not detected by the anti-6×His antibody, confirming earlier reports that it represents a C-terminally processed Rv1860 protein which thereby lost the 6×Histidine tag [16], [27]. Western blotting of rabbit anti-Rv1860 immunoprecipitates with Con-A-HRP (Figure 1C, lower panel) as well as inefficient precipitation of the 45 kDa species from lysates and culture filtrates with Con-A-Sepharose beads (Figure 1D, upper panel), both revealed the paucity of mannosylation on the 45 kDa species as shown earlier by mass spectrometry [16], despite its greater abundance in the lysates of both BCG-TB1860 and BCG-TB1860His as seen in Figure 1A. Specific immunoprecipitation of the 47 kDa mannosylated form of Rv1860 from lysates and culture filtrates of BCG-TB1860His by mouse anti-6× Histidine antibody (Figure 1E, upper panel), which was demonstrated to be glycosylated based on Con-A-HRP reactivity (Figure 1E, lower panel), corroborated these findings. These results showed that the recombinant 6×Histidine tagged protein was glycosylated and secreted similar to the endogenous protein. Additionally, comparison of upper and lower panels in Figure 1 C suggested that glycosylation is significantly higher in the secreted 47 kDa form compared to its counterpart in the lysate both in BCG-GFP and in BCG-TB1860His, in keeping with the translocation-mediated mycobacterial protein glycosylation model proposed by VanderVen *et. al.* [39]. In order to query the surface localization of Rv1860 protein, we carried out staining of the BCG strains with antibodies specific to Rv1860 and the 6×Histidine tag followed by flow cytometry. While the anti-Rv1860 serum detected a 2.2 fold increase in percentage of bacteria with positive surface staining for the protein in BCG-TB1860His relative to BCG-GFP (2.600±0.2082 N = 3% versus 1.200±0.1155%; Figure S3 in Text S1) the mouse Anti-6×Histidine-specific monoclonal antibody did not detect surface expression of the Rv1860-6His protein by flow cytometry (data not shown). We were therefore unable to confirm the surface localization of Rv1860-6His. We also observed that both BCG-GFP and BCG-TB1860 multiplied at comparable levels when grown in liquid 7H9 broth (Figure 1F).

### Expression of Rv1860 of MTB in BCG abrogates the protective efficacy of BCG in the guinea pig animal model

**Figure 2:**
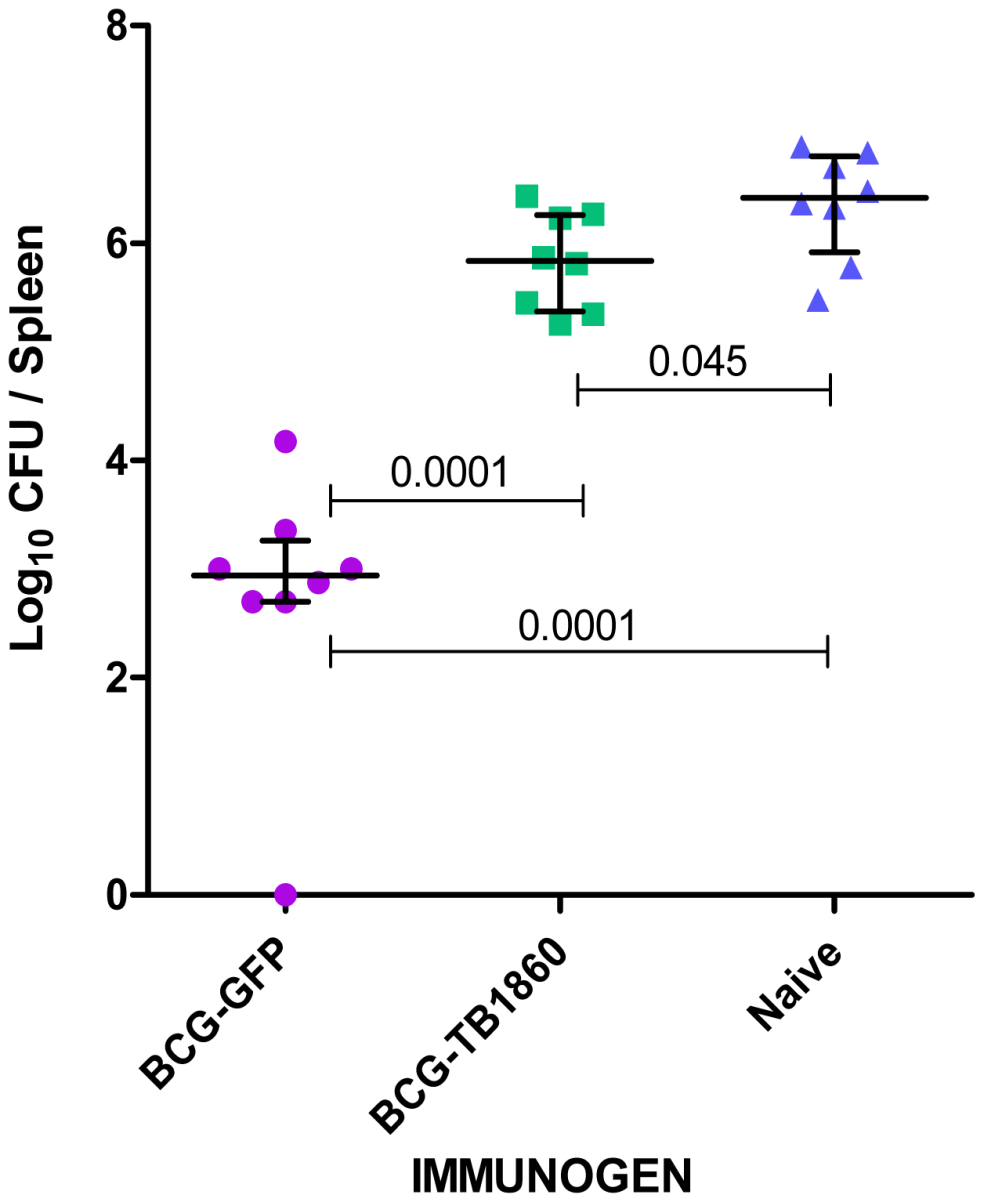
MTB Rv1860 abrogates the protective efficacy of BCG. The Rv1860 gene of MTB was inserted into the genome of BCG-SSI to give BCG-TB1860, as described in Methods. Groups of eight guinea pigs immunized once with 10^6^ viable BCG-GFP and BCG-TB1860 strains were challenged by the intramuscular route with 2×10^5^ viable virulent MTB field strain NTI83949. Challenge bacterial burden in the spleens of guinea pigs six weeks post challenge was enumerated. Naïve control animals received saline injection.

It was the impressive protective efficacy of a DNA vaccine and a poxvirus recombinant expressing Rv1860 of MTB [31], that encouraged us to construct the above BCG recombinant expressing MTB Rv1860 from a copy of the gene inserted into the mycobacteriophage L5 integration site of BCG. As shown above, BCG carries in its genome the homologue of Rv1860 with a total length identical to that in MTB, of 325 amino acids but with a single amino acid difference at residue 140 which is phenylalanine in MTB and leucine in BCG. To our surprise, guinea pigs immunized with the recombinant BCG-TB1860 failed to control bacterial burden from a challenge dose of virulent MTB, with bacterial counts six weeks post challenge reaching those of unvaccinated controls (Figure 2). Thus, BCG expressing MTB Rv1860 sufferred loss of its protective efficacy.

### Rv1860 from MTB inhibits secretion of cytokines by BCG-infected dendritic cells

**Figure 3:**
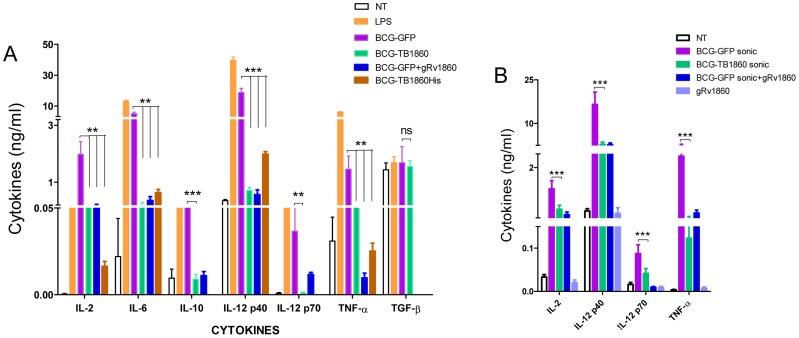
Expression of MTB Rv1860 in BCG suppressed cytokine secretion from mouse BMDC. (A) BALB/c bone marrow differentiated for 7 days with GM-CSF into dendritic cells were infected at 5 bacteria per cell of the indicated BCG strains. Culture supernatants were harvested 8 hrs later and used for cytokine measurements. IL-12p70 and IL-10 were not determined for BCG-TB1860His. Values are mean ± SD obtained from five mice. (B) BALB/c BMDC were treated with sonicated preparations of the BCG strains indicated, comparable to infection at 5 MOI. Glycosylated native Rv1860 (g1860) was added at 10 ng/well, estimated to be consistent with to that coming from 5 MOI infection as described in Methods. Cytokines were measured after 8 hrs of incubation. Values are mean ± S.D. from minimum of 3 mice.

We then queried the mechanism by which the Rv1860 protein of MTB abrogates protective immunity elicited by BCG. Owing to the glycosylated nature of native Rv1860, we surmised that this glycoprotein perhaps interfered with cells of the innate immune system. Indeed, compared to BMDC infected with BCG-GFP, levels of pro-inflammatory and regulatory cytokines including IL-2, IL-6, IL-12p40, IL-12p70, IL-10, and TNF-α secreted by BMDC infected with BCG-TB1860 at 5 MOI, were significantly reduced (Figure 3A), with reproducible reduction of 80 to 95% in secreted cytokine levels, while that of TGF-β, a well-established immune regulatory cytokine [40], [41] was unaltered. BCG-TB1860His infection also resulted in similar reduction of IL-2, IL-6, IL-12p40 and TNF-α (Figure 3A). The loss of IL-2, IL-6 and TNF-α was nearly complete in several experiments despite variation among mice observed as reported by other workers also [42]. IL-12p40 showed the least inhibition, with BCG-TB1860-infected DC still secreting as much as 20 to 30% of the levels produced by DC infected with BCG-GFP (Figure 3A). Interestingly, we observed reduction also in the low levels of IL-10 secreted by BMDC infected with BCG-TB1860 (Fig. 3A) compared to that induced by BCG-GFP, although this cytokine has been reported to have an antagonistic effect on IL-12 secretion by DC infected with MTB [8], [43]. Sonicated preparations of the BCG strains added to BMDC at levels comparable to live infection at 5 MOI resulted in secretion of identical levels of cytokines (Figure 3B), suggesting that MTB Rv1860 mediated inhibition of cytokine secretion by binding to a cell surface receptor. As expected therefore, addition of purified glycosylated Rv1860 (g1860; Table 1) of MTB to BMDC cultures treated with live BCG-GFP or sonicates thereof also resulted in inhibition of cytokine secretion to levels observed following infection with live BCG-TB1860 (Figure 3, A and B), while g1860 added alone had no effect on BMDC, reminiscent of the lack of effect of LAM alone on human blood monocyte derived DC [19]. Non-glycosylated *E. coli*-expressed recombinant Rv1860 added alone or with live/sonicated BCG-GFP had no effect on BMDC (data not shown).

### Rv1860 from MTB inhibits upregulation of cell surface co-stimulatory molecules by dendritic cells

**Figure 4:**
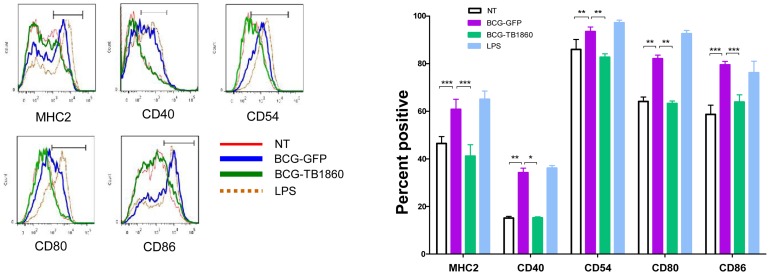
(A). Flow cytometry analysis of surface co-stimulatory molecules on infected BMDC. BMDC were infected with the indicated BCG strains at 5 MOI. 24-FITC and PE- conjugated antibodies specific to the indicated surface co-stimulatory molecules. Plots show the histogram for each marker on CD11c-high gated cells. Key describes the identity of each line. Data were analyzed using Flow Jo software. Values are mean ± S.D. from 3 mice. (B). Data obtained from (A) for percentage of cells positive for each co-stimulatory marker mentioned below were analyzed using Graph Pad Prism software. P values for significant differences between the two strains of bacteria are indicated: *, p<0.05; **, p<0.01; ***, p<0.001.

The ability of dendritic cells to prime an adaptive T cell immune response is critically dependent on the cognate interaction between the co-stimulatory molecules such as MHC-II, CD40, CD80 and CD86 on the APC and the corresponding ligand on the T cell [44]–[46]. We therefore asked if expression of Rv1860 would adversely affect the capacity of BCG-infected DC to up regulate surface expression of co-stimulatory molecules. While BCG-GFP brought about a dramatic increase in the surface expression of CD40, CD54, CD80, CD86 and MHC-II (Figure 4A, blue line), infection of BMDC with BCG-TB1860 (Fig. 4A, green line) drastically reduced the expression of all five co-stimulatory molecules studied, to levels comparable to that found on uninfected DC (Fig. 4A, red line). The reduction was found to be significant for all the markers analyzed (Figure 4B). We did not observe significant variation in CCR7 expression on DC infected with these BCG strains (data not shown). Robust response of BMDC to LPS was observed as expected (Fig. 4A, brown dotted lines).

### Rv1860 from MTB negates the ability of BCG-infected DC to polarize T cells in the TH1 direction

**Figure 5:**
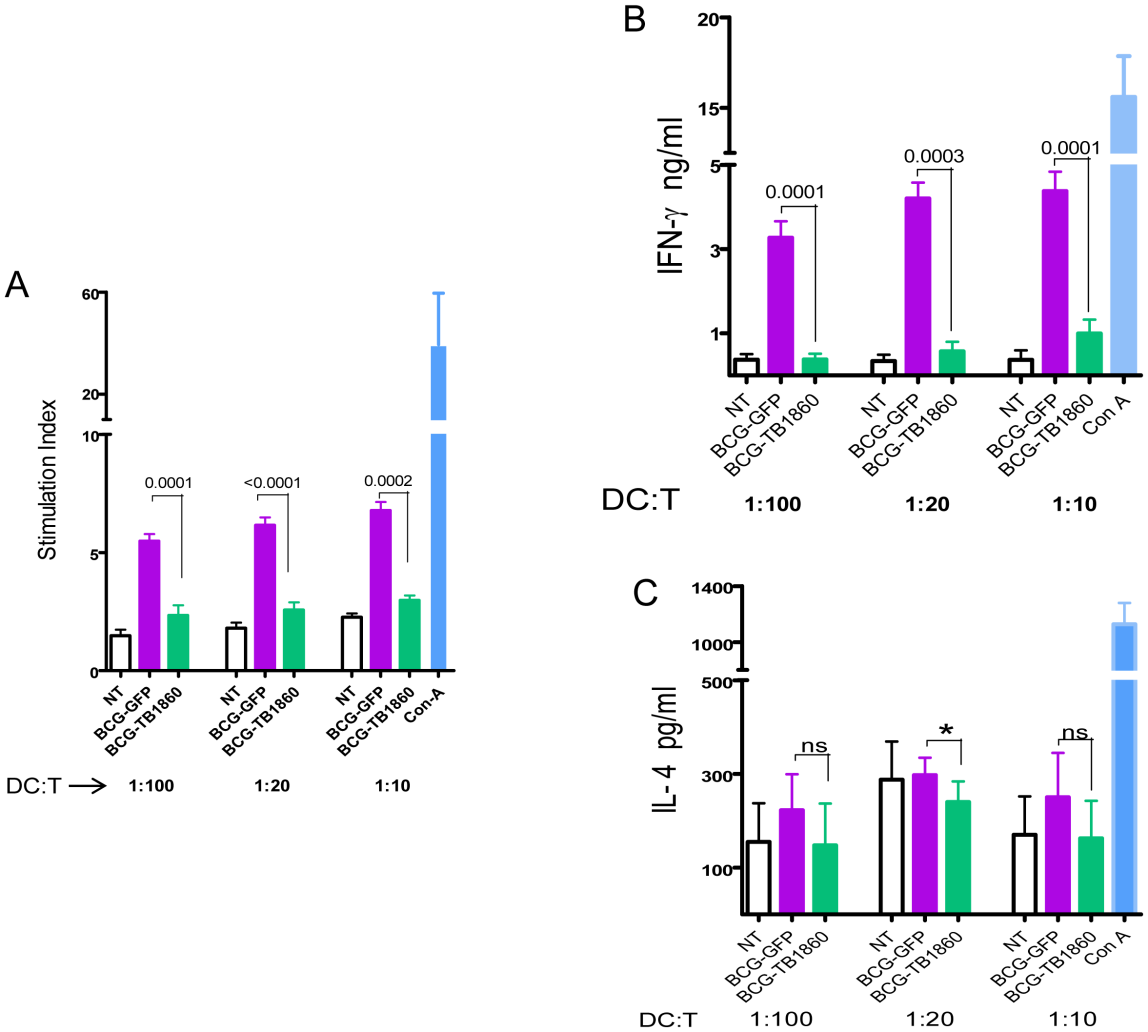
Polarization of allogeneic splenocytes by BCG-infected BMDC. 7 day differentiated BALB/c BMDC were either untreated (NT) or infected with the indicated BCG strains at 5 MOI for 24 hrs, gamma irradiated as described in methods section and co-cultured with C57/Black6 splenocytes at T cell/DC ratios of 10, 20 and 100 for 72 hrs. Supernatants were then collected for measurement of IFN-γ (B) and IL-4 (C). Cells were pulsed with 0.5 µCi tritiated thymidine for 16 hrs, harvested and counted. Stimulation indices (A) were obtained by dividing cpm obtained for each stimulation by sum of cpm obtained for corresponding DC plus T cells alone. Values are mean ± S.D. from at least four mice.

Dendritic cells possess the unique ability to prime naïve T cells and activate them to mature into effector T cells [11], mediated by secretion of inflammatory cytokines in combination with the cognate interaction with T cells through the upregulated surface molecules. Having observed the reduction in cytokine secretion as well as surface co-stimulatory molecule expression by DC infected with BCG-TB1860, it was logical to ask if such DC showed impaired ability to polarize T cells towards a TH1 cytokine profile, a vital prerequisite for establishing protective immunity against MTB. In allogeneic MLR assays, BALB/c BMDC infected with BCG-GFP elicited significant proliferation of and IFN-γ secretion from C57Black6 splenocytes (Figure 5, A and B) whereas similarly cultured DC infected with BCG-TB1860 failed to induce either proliferation (Figure 5A) or IFN-γ secretion (Figure 5B) from C57BL/6 splenocytes. Interestingly, we did not detect increase in IL-4 secretion from splenocytes cultured with BCG-TB1860 infected DC (Figure 5C). This, coupled with the reduction in IL-10 secretion by DC infected with BCG-TB1860, suggested that this glycoprotein functioned primarily to prevent the initiation of a TH1-polarized adaptive immune response, without causing TH2-polarization.

**Figure 6:**
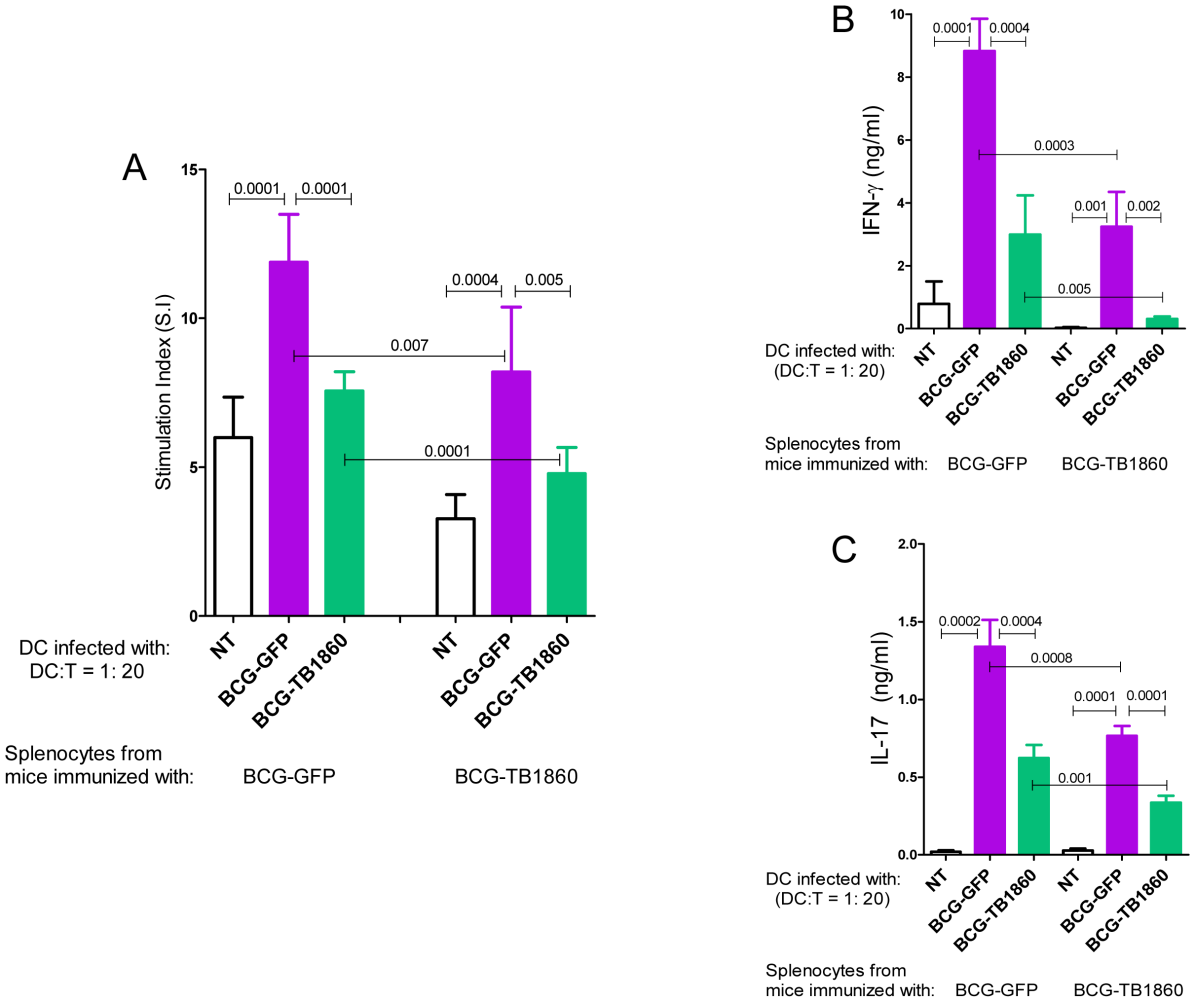
Polarization of syngeneic splenocytes by infected BMDC. 7 day differentiated BALB/c BMDC were either untreated (NT) or infected with the indicated BCG strains at 5 MOI for 24 hrs, gamma irradiated as described in methods section and co-cultured with splenocytes from BALB/c mice infected with 10^6^ viable bacilli of BCG-GFP or BCG-TB1860 3 weeks previously, at T cell/DC ratios of 20 for 72 hrs. Supernatants were then collected for measurement of IFN-γ (B) and IL-17 (C). Cells were pulsed with 0.5 µCi tritiated thymidine for 16 hrs, harvested and counted. Stimulation indices (A) were obtained by dividing cpm obtained for each stimulation by sum of cpm obtained for corresponding DC plus T cells alone. Values are mean ± S.D. from four mice.

**Figure 7:**
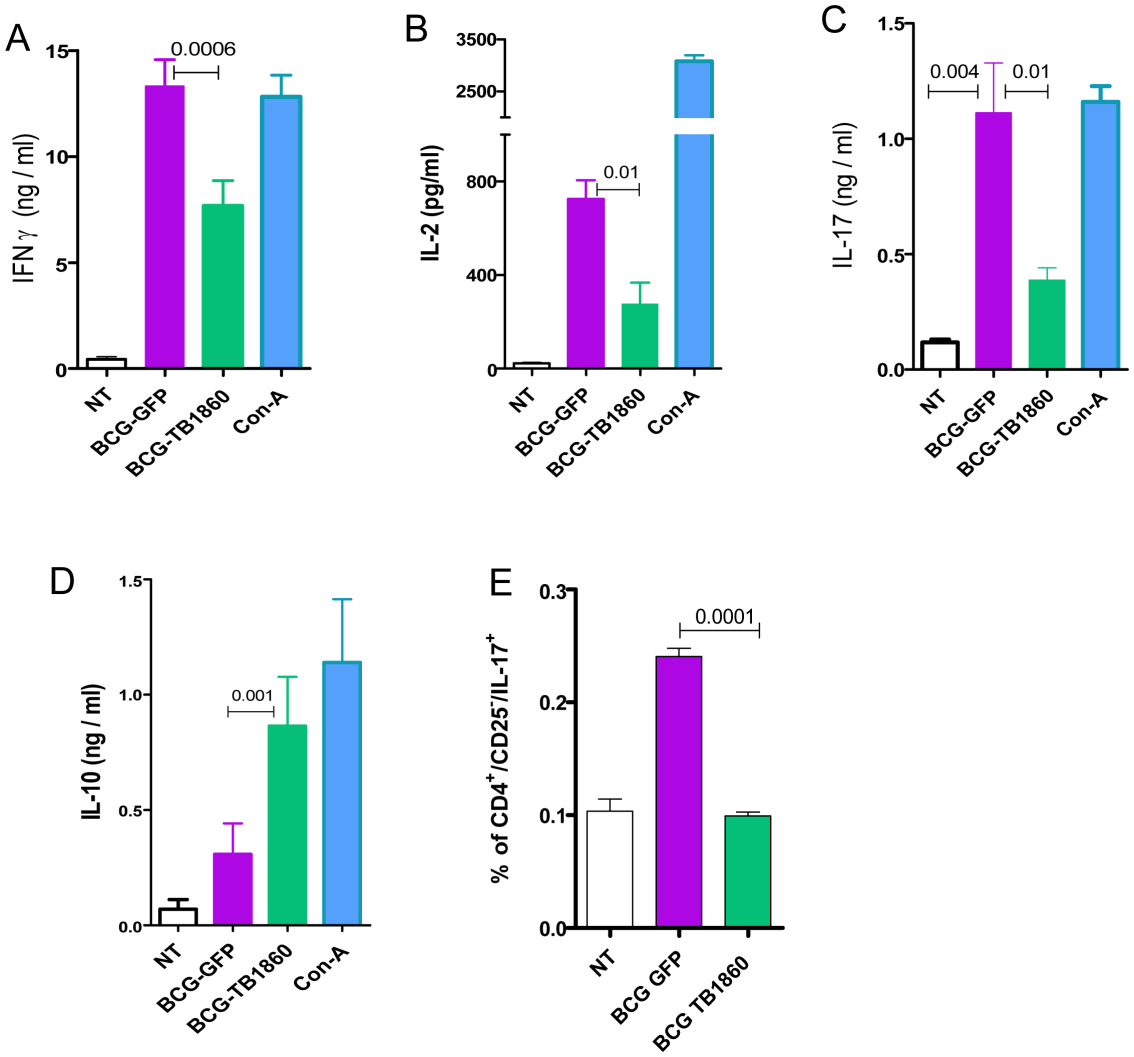
*In vitro* recall responses of splenocytes from BCG-immunized mice. 6 week old BALB/c mice were immunized with 10^7^ viable BCG-SSI. Splenocytes recovered 3 weeks later, were stimulated *in vitro* at an MOI of 20 with BCG-GFP or BCG-TB1860 for 72 hrs. Con-A at 5 µg/ml was included as positive control. Supernatants were collected for the measurement of IFN-γ (A), IL-2 (B), IL-17 (C) and IL-10 (D). (E) Splenocytes stimulated for 36 hrs with the indicated BCG strains were stained for intracellular IL-17. Percentage of cells secreting IL-17 among those gated sequentially on CD3^+^, CD4^+^ and CD25^−^ are displayed. Values are mean ± S.D. from four mice.

In syngeneic polarization assays using splenocytes from mice immunized either with BCG-GFP or BCG-TB1860 also, BMDC infected with BCG-TB1860 inhibited the proliferation (Figure 6A) as well as secretion of IFN-γ (Figure 6B) and IL-17 (Figure 6C) induced by BCG-GFP-infected BMDC. The response of splenocytes from BCG-GFP-immunized mice was significantly higher than splenocytes from BCG-TB1860-immunized mice, to antigen presentation by DC infected *in vitro* with either BCG-GFP or BCG-TB1860 (Figure 6, A to C). We did not detect any changes in levels of IL-4 secreted by splenocytes exposed to the different DC populations (data not shown). In addition, relative to BCG-GFP, BCG-TB1860 inhibited *in vitro* TH1 and TH17 recall responses of splenocytes from BCG-immunized mice. Secretion of IFN-γ, IL-2 and IL-17 by splenocytes from mice immunized with BCG-SSI and infected *in vitro* with BCG-TB1860 were significantly reduced relative to those infected with BCG-GFP (Figure 7, A, B and C) while levels of IL-10 were significantly increased (Figure 7D). Intracellular staining for IL-17 of splenocytes from BCG-immunized mice also revealed a significant reduction in IL-17 secreting CD3^+^CD4^+^CD25^−^ T cells following *in vitro* stimulation with BCG-TB1860 relative to BCG-GFP (Figure 7E).

### Growth and survival of BCG strains in vitro and within BMDC and peritoneal macrophages

Although DC, unlike macrophages do not possess the ability to kill phagocytized bacteria, we asked if the ability to prevent BMDC activation was accompanied by a survival advantage within these two cell types *in vitro* for BCG-TB1860. The bacterial load within BMDC and mouse peritoneal macrophages 3 days post infection with BCG-GFP or BCG-TB1860 were similar; both strains suffered a similar 4 to 5 fold reduction in viable bacteria 72 hrs post infection compared to that at 5 hrs post infection in BMDC (Figure S4A in Text S1). Mouse peritoneal macrophages infected at an MOI of 1 also supported similar 3 fold increase of the two strains over a period of 72 hrs. (Figure S4B in Text S1).

### Effect of Rv1860 on mouse spleen DC *in vivo*

**Figure 8:**
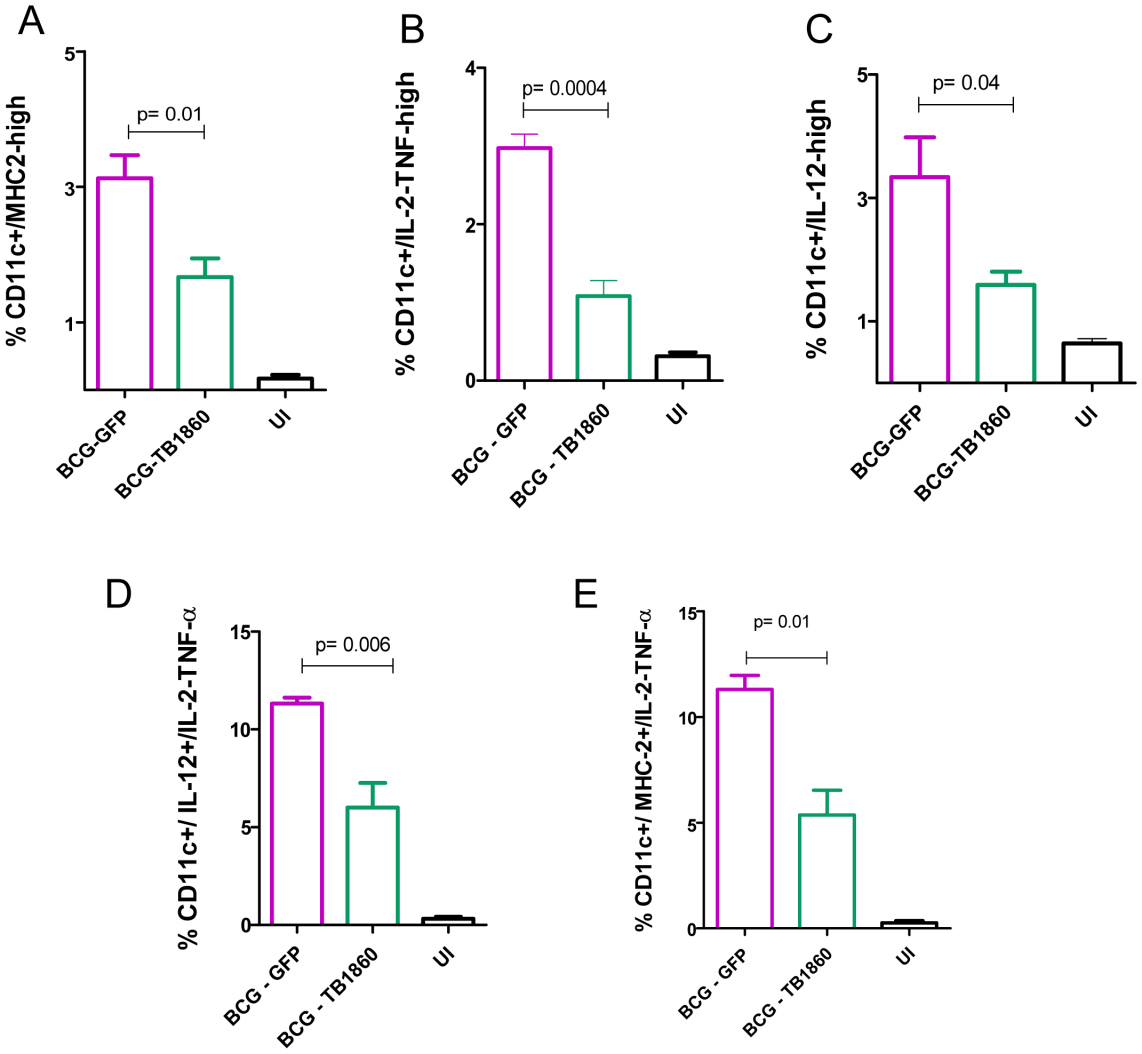
Analysis of spleen DC *ex vivo* from infected mice. Splenocytes obtained from mice uninfected (UI) or infected 12 hours earlier with 10^7^ cfu of BCG-GFP or BCG-TB1860 were processed for flow cytometry as described in Methods and illustrated in Figure S5 in Text S1. The number of CD11c-high DC displaying upregulated expression MHC class II (A), secreting IL-2/TNF-α (B) and secreting IL-12p40 (C) are shown as a percentage of total splenocytes within gate P1 of Figure S5A. Splenocytes simultaneously secreting IL-2/TNF-α along with IL-12p40 (D) or simultaneously secreting IL-2/TNF-α along with expression of MHCII (E) are shown as a percentage of total CD11c-high DC.

We next asked if the *in vitro* effects of BCG-TB1860 infection on BMDC would be corroborated by similarly reduced DC functions *in vivo* in mice infected with BCG-TB1860 relative to BCG-GFP. A spurt of IL-12p40 production was reported by spleen dendritic cells as early as 5 hours following infection of mice with BCG [47]. We analyzed spleen dendritic cells *ex vivo* from mice infected for 12 hrs, for surface expression of MHC class II as well as synthesis of cytokines IL-12, and IL-2/TNF-α by intracellular cytokine staining followed by flow cytometry using the gating strategy shown in Figure S5A in Text S1. We detected significant reduction both in the numbers of DC up-regulating surface MHC-II as well as producing the above mentioned cytokines in splenic DC from mice infected 12 hrs previously with BCG-TB1860 compared to mice infected with BCG-GFP (Figure 8, A, B and C and Figure S5B in Text S1). Significant reduction in DCs that simultaneously expressed a combination of either IL-2/TNF-α and IL-12 or IL-2/TNF-α and MHC-II (Figure 8, D and E and Figure S4C in Text S1) was also observed.

### Identity of Rv1860 receptor on BMDC

We next wished to determine the identity of the pattern recognition receptor (PRR) on DC that bound Rv1860 as ligand. Blocking antibodies to the mouse mannose receptor, Dectin-1 and SIGNR1 added to BMDC prior to infection brought about no alleviation of the inhibition of BCG-GFP-induced cytokine secretion that we observed with BCG-TB1860 (Figure S6 in Text S1). In the same experiment, a TLR-4 blocking antibody resulted in approximately 50% reduction of cytokine secretion elicited by LPS (data not shown), similar to the inhibition reported earlier [48] for this antibody. Control isotype antibody and pre-immune rabbit serum samples did not cause perturbation of the BCG-TB1860-induced inhibition of cytokine production, as expected. The non-availability of commercial blocking antibodies for murine DC-SIGN, the human homologue of which the Rv1860 protein has previously been reported to bind [21], precluded addressing the role of this PRR in mediating the Rv1860 effect on mouse BMDC.

## Discussion

Immune protection against MTB is an extremely complex phenomenon where the multiple cells of the immune system along with their mediators work both in concert and at cross purposes, often succeeding and occasionally (in 5 to 10% of individuals) failing to achieve just the right balance required to prevent disease {reviewed in O'Garra, 2013 #183}. Subversion of cells comprising the innate immune system by MTB has been extensively documented. Macrophages, dendritic cells and neutrophils are all infected by MTB in the mouse model of aerosol infection [5] with resultant subversion of their functions. In this model, DC were demonstrated to be one of the earliest infected population, second only to neutrophils. Following intranasal inoculation of mice with as little as one million BCG, 5% of lung cells were infected by 24 hrs, of which 15% were DC [49], corroborated by another study that demonstrated phagocytosis of BCG by almost 40% of lung DC by 24 hrs post intranasal infection [50]. This latter study also showed the superior production of IL-12 and TH1 polarization of CD4^+^ T cells by recruited DC over alveolar macrophages. Both these studies reproducibly implicated both alveolar macrophages and DC as the predominantly infected cells early in lung infection. Both in infected mouse lung and spleens, the subset of DC infected early were lymphoid [47], [50], replaced by myeloid DC post 2 weeks following infection [5], [47]. DC, despite constituting a mere 6.8% of total lung tissue, were found to represent 50% of BCG-infected cells in lungs by 2 to 3 weeks post low dose aerosol infection [5]. All these studies point to the high propensity of tissue DC to be infected regardless of route of infection by the slow growing mycobacteria. Keeping in mind that immature DC are highly phagocytic, it is not surprising that both *in vivo* and *in vitro*, DC are efficiently infected, not to mention the numerous pathogen recognition receptors that they boast of, many of which such as DC-SIGN and mannose receptor, have already been shown to play a role in internalization of mycobacteria [5], [51], [52].

Mouse BMDC and BMDM cultures infected *in vitro* by MTB have also been extensively used to demonstrate loss or reduction of their normal functions by this pathogen [8], [53]. Sections of infected human lung tissue revealed infection of DC as well as alveolar macrophages by MTB [52], [54] and MTB infection resulted in a differential cytokine response from human monocyte-derived DC and macrophages [55]. The major receptor on human DC used by MTB to gain entry into these cells was demonstrated to be DC-SIGN [19], [52] which recognized the abundant mannose capped lipoarabinomannan (Man-LAM) from the cell wall of MTB as the dominant ligand [19]. This binding resulted in secretion of IL-10 and inhibition of LPS- and *M. bovis* BCG-mediated maturation of DC. Later studies however revealed that human DC-SIGN binds to multiple ligands derived from MTB in addition to Man-LAM, including lipomannan and the mannose-containing 19 and 45 kDa glycosylated proteins [21]. Subsequent investigations using live mycobacteria in the place of purified LAM clearly revealed that LAM makes at best a minor contribution to DC-SIGN binding during infection of human DC by mycobacterial pathogens [20]. One report concluded, based on inhibition by mannan, that Man-LAM binds to the mannose receptor (MR) on human DC [56], resulting in inhibition of IL-12 secretion. However, a later study that used blocking antibodies to MR and DC-SIGN revealed that ManLAM binds to DC-SIGN and not to MR on human DC [57]. Our results demonstrated sizeable loss of multiple BMDC functions both *in vitro* and *in vivo*, including cytokine secretion, up regulation of surface markers and downstream effects on TH1 and TH17 cytokine secretion by *in vitro*-polarized T cells along with significantly reduced cytokine secretion by mouse splenic dendritic cells recruited early, within 12 hrs of BCG infection, suggesting that Rv1860 (along with other glycoproteins of MTB) makes a measurable contribution towards suppressing DC functions very early following infection. That these early events ultimately lead to severely compromised protective immunity, was suggested by the dramatic increase in challenge MTB burden in the guinea pig vaccination model.

We observed a dramatic reduction in the secretion of several cytokines including IL-2, IL-10, IL-12 p40 and p70, and TNF-α by BCG-TB1860-infected BMDC. Early IL-2 secretion by DC was shown to be key for efficient T cell stimulation [58], [59] and IL-2–deficient DCs when activated by bacteria were severely impaired in the ability to induce allogeneic CD8^+^ and CD4^+^ T-cell proliferation compared with wild-type (WT) DCs [58]. BCG-TB1860 infection extinguished IL-2 secretion by BMDC almost completely. The ability of DC to migrate to the draining lymph nodes following encounter with pathogens and subsequent initiation of the T cell response is governed by the cytokines they secrete soon after encounter with pathogens. IL-12 p40 levels have been shown to be critical for DC migration and TH1 T cell priming in MTB infection [55], [60], [61]. The importance of IL-12p70 in resistance to tuberculosis has been convincingly demonstrated [62], [63]. Thus, the significant reduction in BMDC secretion of both IL-12p40 and IL-12p70 caused by Rv1860 poses a threat to effective initiation of a protective TH1-biased T cell immune response against TB. BMDC infected with BCG-GFP secreted low levels of IL-10 (50 to 100 pg/ml, similar to that reported earlier [53] following infection of 5 day cultured C57BL/6 with the Erdman strain of MTB), which was completely extinguished by BCG-TB1860. IL-10 secreted at high concentrations by DC is also known to induce development of tolerogenic and regulatory T cells [64]. The failure of MTB-infected macrophages to secrete IL-12 has in turn been demonstrated to be mediated through IL-10 production, with both DCs and macrophages from IL-10 knockout mice secreting multiple fold more IL-12 p70 compared to wild type mice [8]. The loss of IL-10 from BMDC exposed to MTB Rv1860 suggests that modulation of this cytokine is not one of the mechanisms deployed by Rv1860 to prevent TH1 polarization of T cells.

Expression of co-stimulatory molecules by DC is critical for their ability to deliver the second signal to T cells and activate both CD4^+^ and CD8^+^ T cells. CD80 (B7-1), CD54 (ICAM-1) and LFA-3 were shown to function synergistically to boost proliferation, cytokine secretion and effector functions of T cells [65], [66] while the regulated recruitment of CD86 to lipid rafts on DC was shown to be essential to achieve optimal T cell activation [67]. Ligation of CD40 on human DC was also shown to result in the dramatic up regulation of the surface co-stimulatory molecules CD80 and CD86 [68] and to serve as efficient stimulus for IL-12 production and subsequent TH1 polarization of T cells [69]. Adoptively transferred naïve antigen-specific CD4+ T cells lacking CD40-ligand failed to divide when the recipient mice were challenged with antigen [44] which could be attributed to their inability to engage CD40 on antigen-presenting DC and induce them to secrete IL-12. CD40-CD40 ligand interaction is also vital for generating TH17 T cells [70]. It is therefore striking that Rv1860 of MTB was capable of suppressing a wide range of co-stimulatory molecule expression on DC to levels found on uninfected cells. These *in vitro* effects on BMDC and splenocytes were replicated during infection of mice by these BCG strains, as evidenced by the depressed activation of spleen DC at early time points after infection of mice. Surface expression of MHCII as well as secretion of IL-2, IL-12 and TNF-α were all compromised when spleen DC were analyzed *ex vivo*.

The results we report here have significant implications beyond innate immune responses, for the generation of a TH1- and TH17-biased adaptive cell mediated immune response that is vital for protection against TB [71], [72]. In keeping with the significantly reduced secretion of inflammatory and regulatory cytokines and loss of surface co-stimulatory molecules by BCG-TB1860-infected BMDC, splenocytes stimulated with these DC suffered almost complete loss of ability to secrete IFN-γ, a cytokine that again has been shown to be vital for anti-TB immunity [73], [74] as well as IL-2 and IL-17. Rv1860 did not affect the levels of TGF-β secreted by BMDC while significantly reducing the IL-6 levels produced. The combination of these two cytokines is required to generate TH17 cells while TGF-β alone causes polarization of the common precursors to regulatory T cells (Treg) [75], [76]. Selective loss of IL-6 without decrease in TGF-β as seen following BCG-TB1860 infection of BMDC would therefore polarize precursors to differentiate into Treg at the expense of TH17. In fact, we did observe significant decrease in the percentage of IL17-secreting CD3^+^CD4^+^CD25^−^ T cells and a significant increase in splenocyte secretion of IL-10, a cytokine secreted by Treg cells, following infection with BCG-TB1860, compared to BCG-GFP. IL-10 is elevated in human TB disease [77] and has been shown to prevent fibrotic granuloma formation and initiation of CD4^+^ T cells with a TH1 profile in the mouse model of TB {Cyktor, 2013 #161}. Splenocytes from BCG-TB1860-infected mice could not regain their TH1 and TH17 cytokine secretion to levels seen in BCG-GFP-immunized mice despite *in vitro* exposure to high MOI of BCG-GFP or to BCG-GFP-infected BMDC, revealing a long lasting deleterious effect of Rv1860 on the ability of dendritic cells to initiate a TH1 and TH17-dominated T cell response, reflecting the loss of TH1 and TH17 cells observed in human TB disease [72].

The guinea pig animal model provided convincing evidence that this subversion of DC function and subsequent TH1 T cell activation by Rv1860 had far reaching deleterious effects on protection afforded by BCG. BCG-immunized mice splenocytes stimulated *in vitro* with BCG-TB1860 would model the effect of MTB infection in BCG-vaccinated individuals and perhaps explain the recorded failure of BCG vaccination against adult pulmonary TB [78] in India and Africa. That MTB actively delays the initiation of adaptive immunity is well documented [79], shown to be mediated through delayed migration of infected DC from the sites of infection to the draining lymph nodes [80]. Our results suggest that the impairment of DC functions reported in these earlier studies may have a contribution from glycoproteins such as Rv1860 through binding of DC receptors.

The mycobacteriophage L5 integration locus within the glycyl tRNA (*glyV*) locus of BCG and MTB is the most widely used site for integrating foreign genes into these slow growing mycobacteria [81]. The Rv1860 protein expressed from the integrated copy of the MTB gene was expressed at similar levels to and was secreted and glycosylated, also similar to the native protein. Similar to previous recombinant strains of BCG carrying integrations, we too did not observe any alteration in growth of BCG-TB1860 relative to BCG-SSI or BCG-GFP. BCG-TB1860 brought about its suppressive effects on DC without exhibiting growth advantage, either in axenic cultures or within *in vitro*-infected BMDC and peritoneal macrophages. This was surprising but leaves open the possibility that BCG-TB1860 indeed survives better than BCG in infected animals *in vivo*, thanks to the presence of the Rv1860 protein. This crucial issue that we were unable to address in this study will remain the focus of our ongoing investigations to understand the mechanisms by which Rv1860 co-opts/subverts host innate immune cells for the benefit of the pathogen.

The receptor on innate immune cells to which Rv1860 binds, to bring about the inhibition of DC functions that we report here awaits identification. Owing to non-availability of commercial blocking antibodies for murine DC-SIGN, we were unable to address this question in its entirety. Indeed, the 45 kDa glycoprotein Rv1860 has been reported to bind several C-type lectins on human innate immune cells, including DC-SIGN [21] and the pulmonary surfactant protein (PSP)-A [33]. Recent studies [13], [14] have identified a vast array of mannosylated proteins in MTB. Whether alpha 1→2 linkages, which are unique to Rv1860 and are required for binding to DC-SIGN [82], are shared by any of these other mycobacterial glycoproteins, and if so, their contribution to pathogenesis of this organism is worth investigating.

*M.bovis*-BCG also carries the gene for and expresses the homologue of Rv1860 which differs from the MTB protein by a single amino acid at residue 136 which is phenylalanine in MTB and leucine in BCG. Despite conservation of the threonines that are glycosylated, the MTB protein is extremely potent at inhibiting DC functions, in contrast to the BCG homologue. The structural motifs that functionally distinguish the MTB Rv1860 from the BCG homologue are currently not known although they do not appear to reside within the glycosylation pattern, since the MTB Rv1860 in BCG-TB1860 is glycosylated by the BCG enzymatic machinery. Thus, the possible role of F136 in the MTB Rv1860 protein in affecting the folding of Rv1860 and its consequent binding to its cognate receptor on BMDC deserves investigation.

The existence of numerous individuals living in TB-hyper endemic regions with no reactivity in the purified protein derivative (PPD) skin test despite continual exposure to MTB [83] attests to the remarkable ability of the innate immune response to control infection by MTB to a degree that prevented T cell activation. In light of the recent demonstration that BCG immunization results in long lasting beneficial epigenetic alterations in monocytes [84], it would be worthwhile to investigate the effect of Rv1860 on chromatin remodeling in cells of the innate immune system. The suppressive effect of Rv1860 on BMDC would point to the exciting possibility of developing efficacious vaccines against TB, based on removal of genes encoding such immune suppressors from the genome of BCG/MTB.

## Supporting Information

Text S1 Supporting information. This file contains Supplementary Methods and Figures S1-S6 with legends.
(DOC)
