# Correction: The Glycosylated Rv1860 Protein of *Mycobacterium tuberculosis* Inhibits Dendritic Cell Mediated TH1 and TH17 Polarization of T Cells and Abrogates Protective Immunity Conferred by BCG

**DOI:** 10.1371/journal.ppat.1004352

**Published:** 2014-08-05

**Authors:** The PLOS Pathogens Staff

There is one error each in the Materials Methods and Results sections, respectively.

1. In the Materials and Methods section, under the subheading "Infection of mice with BCG strains," in the last sentence, "Figure S4" should be "Figure S5A in Text S1".

2. In the Results section, under the subheading "Expression of Rv1860 of MTB in BCG abrogates the protective efficacy of BCG in the guinea pig animal model" in the second sentence, "residue 140" should be "residue 136".
