# Supplementary material for: The Glycosylated Rv1860 Protein of Mycobacterium tuberculosis Inhibits Dendritic Cell Mediated TH1 and TH17 Polarization of T Cells and Abrogates Protective Immunity Conferred by BCG
Source: PLoS Pathog. 2014 Jun 12;10(6):e1004176. doi: 10.1371/journal.ppat.1004176 (PMC4055742; doi:10.1371/journal.ppat.1004176)

**Supplementary Methods**

## **Cloning, expression, purification of recombinant MTB Rv1860 protein and antibody generation.**

## Rv1860 gene of MTB was amplified from genomic DNA of the clinical isolate NTI83949 of MTB using the following forward and reverse primers: 5’ GTGACCATGGCTAGCCATCAGGTGGACCCCAACTTGACACG (FP1 in Table 2) and 5’GTGAGGATCCTCAGGCCGGTAAGGTCCGCTGCGGTGT (RP1 in Table 2), digested with BamHI, Klenow filled, then digested with NcoI, followed by ligation to pET20b cut with XhoI, Klenow filled and NcoI digested. Rv1860 protein present in the soluble fraction of lysates from E. coli harbouring pET20b-Rv1860, induced with 0.1mM IPTG were purified using Ni-NTA resin binding followed by elution using a gradient of 50 to 500 mM imidazole using a GE healthcare EKTA basic-10 FPLC machine. Pooled Ni-NTA eluted fractions diluted 25 fold with 20 mM Tris pH7.8 was applied to a monoQ column, washed with 50 ml of 50mM Nacl 20 mM Tris pH 7.8 and eluted with a 0.05 to 1M linear gradient of NaCl. Fractions containing Rv1860 were pooled, concentrated by ultrafiltration and applied to a Sephadex G-200 gel filtration column developed with phosphate buffered saline. Endotoxin was removed by extracting the protein solution thrice with 1% tritonX-114. Absence of endotoxins in the purified protein preparations was ascertained using the Pyrogent plus® Gel-clot LAL test kit (BioWhittaker Inc., Walkersville, MD). Protein emulsified with incomplete Freund's adjuvant was injected intraperitoneally into mice (10 µg protein per mouse) thrice at 3 week intervals using antigen suspended in PBS. Mice were bled 10 d later by intraocular puncture. Rabbits were immunized thrice 3 weeks apart with 200 µg of recombinant Rv1860 protein emulsified in incomplete Freund’s adjuvant and bled 1 week after the 3^rd^ immunization.

**Plasmid construction.**

We first generated a new mycobacterial integration vector containing a hygromycin resistance gene flanked by the γδ *res* sites as follows: We digested the plasmid pDK10 [83], a kind gift of Anil Tyagi, Delhi University, New Delhi) containing the mycobacteriophage L5 *att-int* sequences with *Dra*I and *Nco*I to remove the ampicillin resistance marker and the lacZ gene, and recovered the Klenow-filled and dephosphorylated 3.2 kb long blunt vector fragment. The hygromycin resistance gene flanked by the γδ *res* sites was obtained from the plasmid pYUB854 , a kind gift of William Jacobs, Jr., Albert Einstein College of Medicine, Bronx, New York} as a blunt fragment by digestion with *Xba*I and *Xho*I followed by Klenow filling and ligated to the 3.2 kb pDK10-derived vector mentioned above to generate pDK-Hyg-res. The plasmid pBEN containing the GFPmut3 gene driven by the *M. bovis* BCG hsp60 promoter was a kind gift of Dr. Lalitha Ramakrishnan, Washington University, Seattle. The Rv1860 gene of MTB obtained by PCR amplification of H37Rv DNA with primers FP1 and RP1 (Table 2) was digested with NcoI, Klenow filled and digested with *Bam*HI followed by ligation downstream of the hsp60 promoter of pBEN cut with *Hind*III, Klenow filled and *Bam*HI digested, to create pBEN-TB1860. The hsp60 promoter-Rv1860 fragment was then excised out of pBEN-TB1860 using *Xba*I and *Xho*I, Klenow filled and ligated to pDK-Hyg-res cut with *Not*I and Klenow filled, to generate pDK-Hyg-Rv1860 (Figure S1 in Text S1). We also constructed pDK-Hyg-GFP containing the hsp60 promoter-driven GFPmut3 gene in pDK-Hyg-res by excising an *Xba*I-*Sal*I fragment from pBEN and ligating to *Not*I-digested pDK-Hyg-res as blunt fragments.

To construct Rv1860 carrying a C-terminal 6X Histidine tag, forward primer 5’ TTTTTTTT GGA TCC ATG CAT CAG GTG GAC CCC AAC TTG 3’ with a *Bam*HI site and reverse primer 5’ TTTTTTTT GTT AAC TCA GTG GTG GTG GTG GTG GTG GGC CGG TAA GGT CCG CTG C 3’ with 6X Histidine tag and *Hpa*I site (FP2 and RP2 in Table 2) were used to amplify the MTB Rv1860 gene, cut with BamHI and HpaI and ligated to similarly digested pBEN to generate pBEN-TB18606His. The Rv1860 gene with hsp promoter and C terminal 6X Histidine tag was released with *Dra*I *and Ssp*I and ligated to pDK-Hyg-res digested with NotI and Klenow filled. All recombinant plasmids were confirmed by sequencing. The orientation of the Rv1860 gene in pDK-Hyg-Rv1860 and pDK-Hyg-Rv1860-6His were opposite to each other.

**Legends for Text S1**

Figure S1 in Text S1. The strategy for construction of pDK-Hyg-Rv1860, and pDK-Hyg-Rv1860-6His expressing the MTB Rv1860 gene without and with a C-terminal 6X Histidine tag downstream of the *hsp* promoter of pBEN {Valdivia, 2000 #38} is shown. pDK-Hyg-GFP was constructed similarly by obtaining the XbaI-XhoI Klenow blunt hsp promoter-GFP fragment from pBEN and ligating it to pDK-Hyg-res.

Figure S2 in Text S1 . Polymerase Chain Reaction (PCR) amplification of genomic DNA from the strains of BCG indicated above the lanes. Lanes 3 to 6 show the endogenous Rv1860 gene amplified using FP3 and RP3 primers to give a 1030 bp product (Table 2); lanes 8 to 11 show the integrated copy of Rv1860 amplified using primers FP4 and RP4 to give a 1500 bp product; Lanes 13 to 16 show the inserted GFP gene amplified using primers FP5 and RP4 to give a 1200 bp product; lanes 19 to 22 show the inserted Rv1860-6XHis gene amplified using primers FP6 and RP4 to give a 1800 bp product. Numbers on the left with arrows refer to the size markers in kilobases.

Figure S3 in Text S1. Analysis of surface localization of Rv1860 by flow cytometry. (A) BCG-GFP (lower left panel) and BCG-TB1860His (lower right panel) were stained with mouse anti-Rv1860 serum followed by goat anti-mouse secondary antibody-APC conjugate. Upper right panel shows the isotype control. Upper left panel shows the scatter plot and gated cells for analysis. Samples were acquired on a BD FACS Canto cytometer. (B) Quantitation of positively staining cells obtained from (A). P value for significant increase in percentage of stained cells in BCG-TB1860His compared to BCG-GFP is shown.

Figure S4 in Text S1. Growth of of BCG strains within infected BMDC and mouse peritoneal macrophages. BMDC (A) and mouse peritoneal macrophages (B) were infected at an moi of 1 with BCG-GFP or BCG-TB1860. Viable bacterial counts recovered at 5 hrs and 72 hrs are shown. Values are mean ± S.D. from 3 mice. ns= not significant.

Figure S5 in Text S1. Gating strategy for intracellular cytokine staining of mouse spleen dendritic cells. Samples acquired on a BD-FACS Canto flow cytometer were analysed using Flow Jo software (Treestar). (A) Singlets gated as shown were displayed on a forward versus side scatter and total splenocytes were displayed on a APC versus side scatter dot plot. The APC-low population devoid of T and B cells, macrophages and neutrophils were gated and displayed on a CD11c versus MHC2/IL12 (PE) or IL-2+TNF-α (PE-Cy7) as shown in B. (C) CD11c-high DC expressing simultaneously a combination of IL-2/TNF-α along with IL-12 or MHCII are shown.

Figure S6 in Text S1. Effect of receptor-blocking antibodies on cytokine secretion by infected BMDC. The secretion of TNF-α and IL-12 p40 was measured following infection of BMDC at moi of 5 with BCG-GFP and BCG-TB1860. The percent inhibition of cytokine secretion brought about by BCG-TB1860 relative to BCG-GFP in the absence (open bars) of presence of blocking antibodies with specificities indicated in the accompanying key is shown. Data are mean ± S.D. from 3 mice.


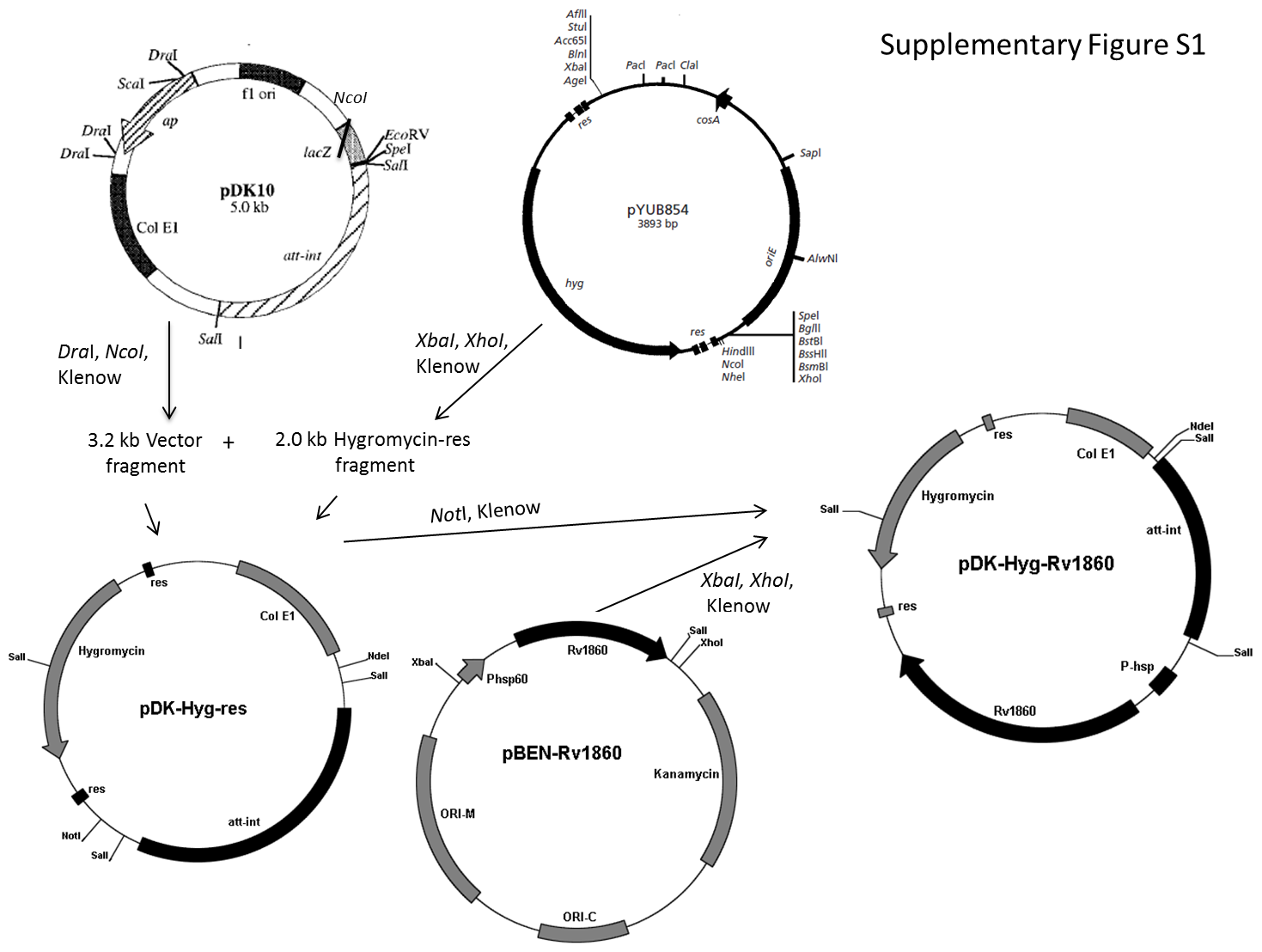


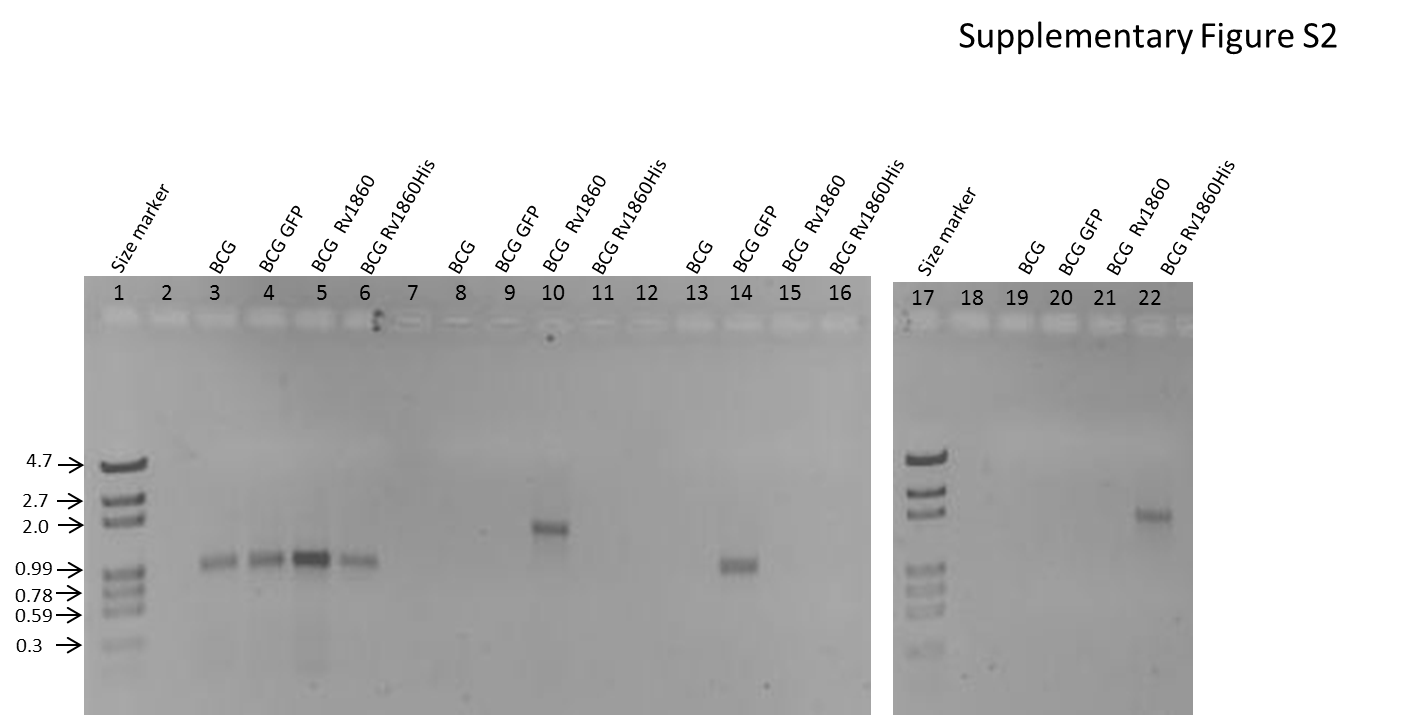


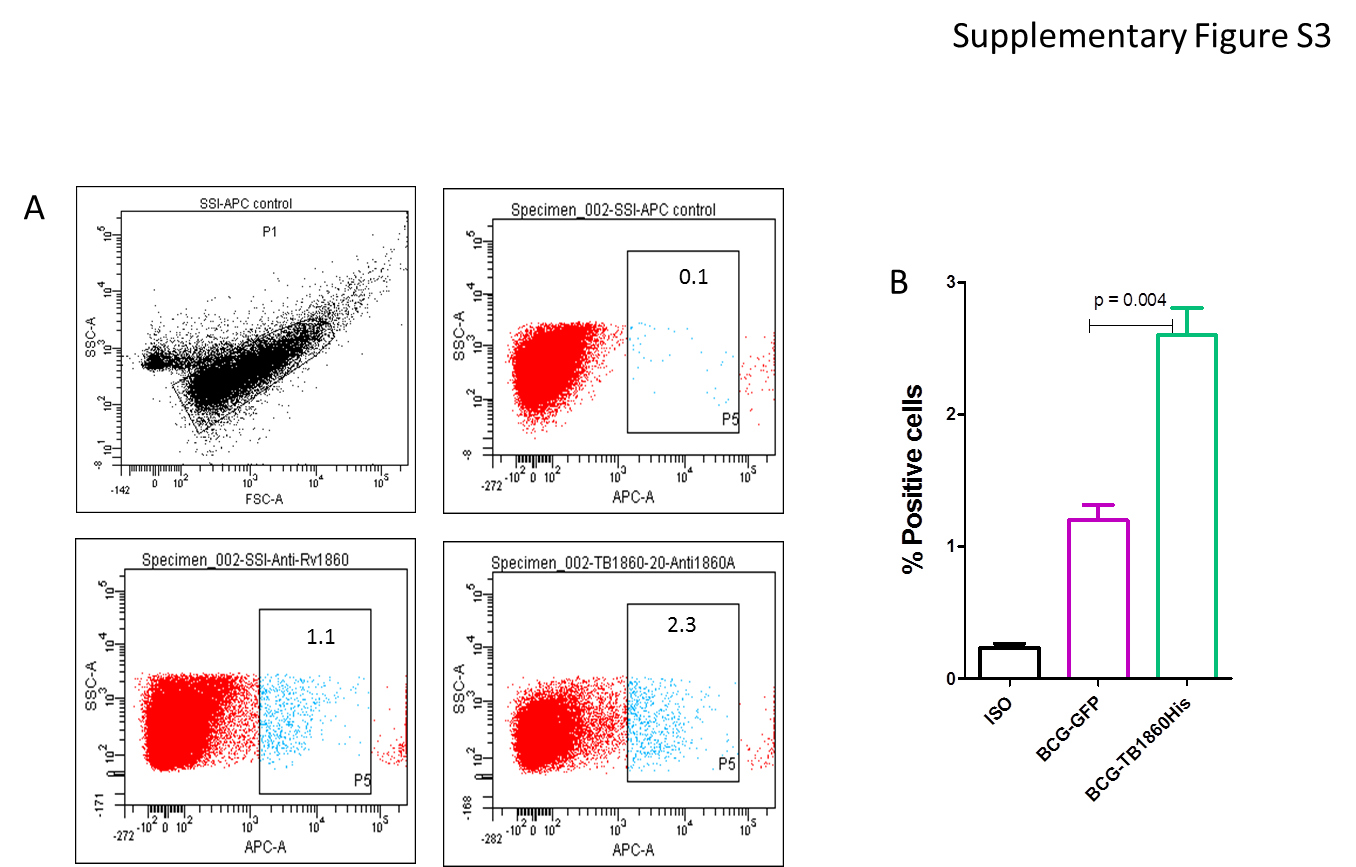


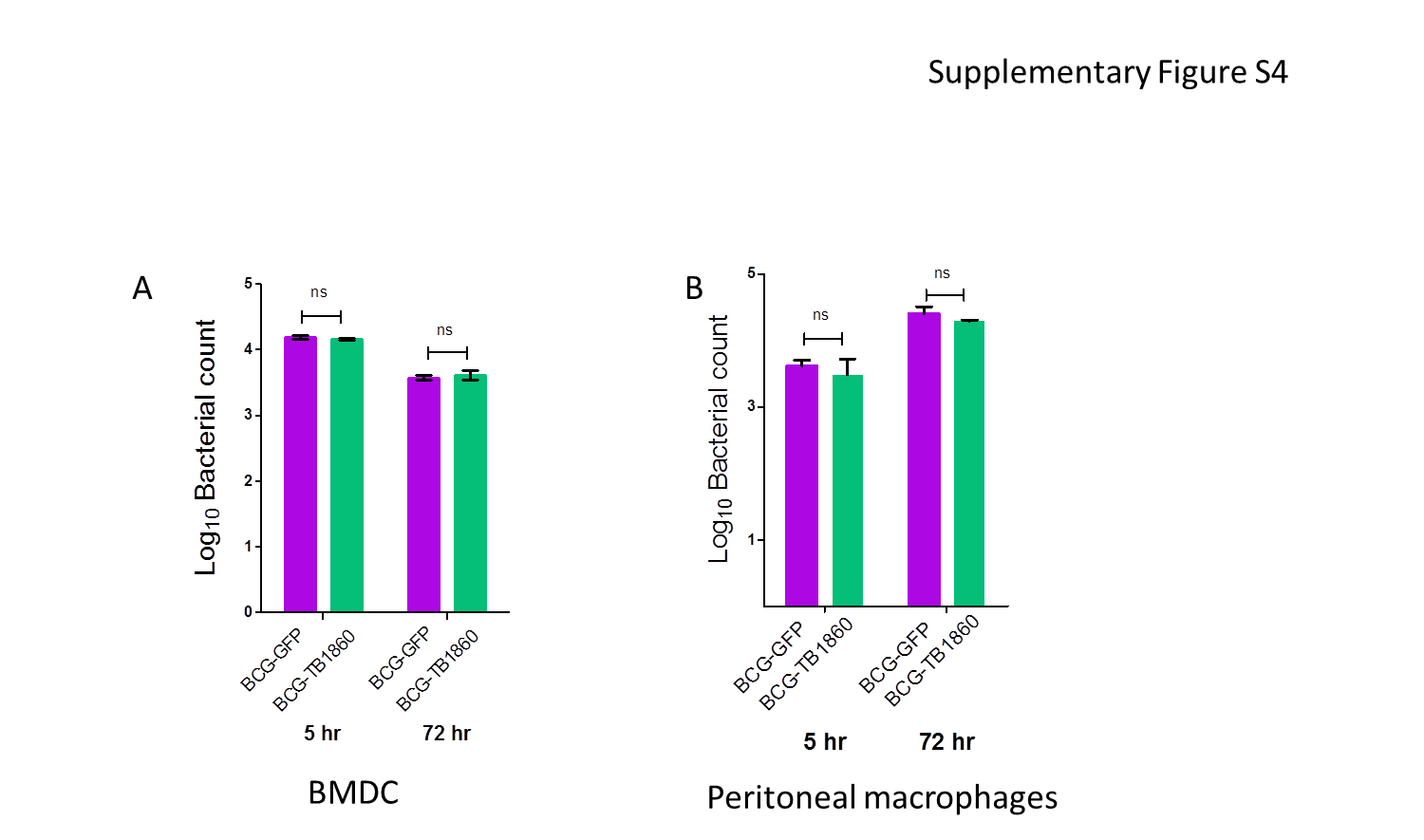


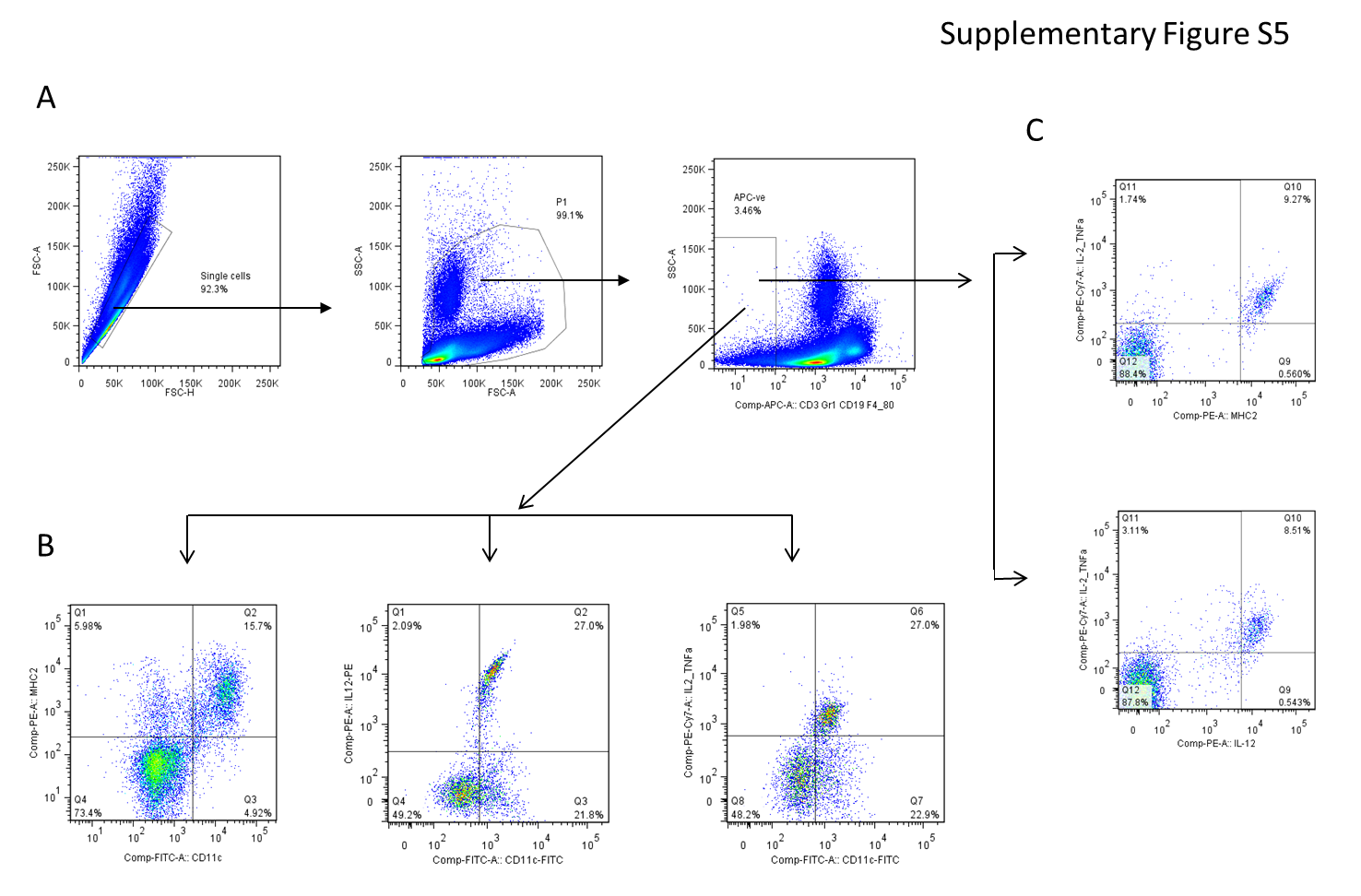


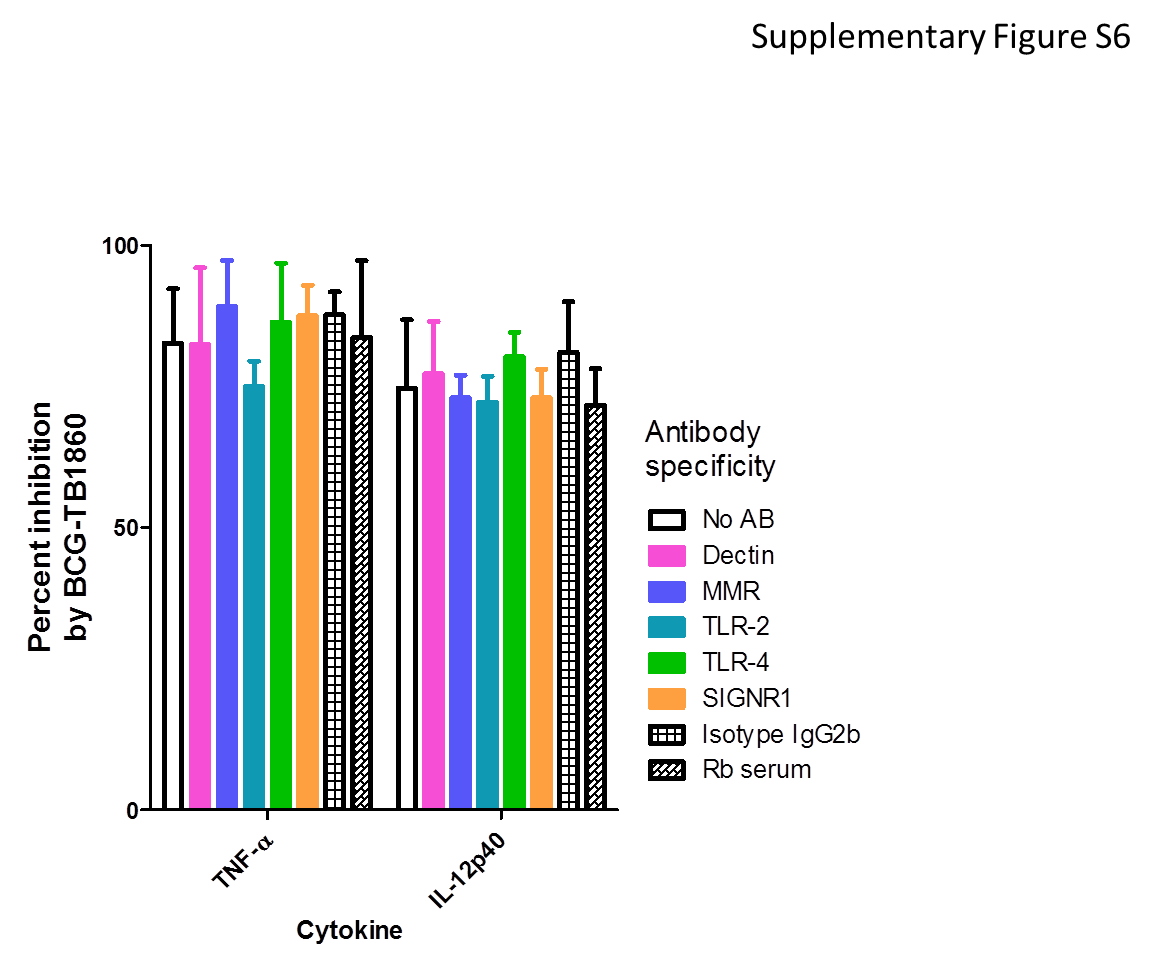

Supplement: Text S1 — Supporting information. This file contains Supplementary Methods and Figures S1-S6 with legends. (DOC) [file ppat.1004176.s001.doc]
